# Deciphering the immune reaction leading to spontaneous melanoma regression: initial role of MHCII^+^ CD163^−^ macrophages

**DOI:** 10.1007/s00262-023-03503-6

**Published:** 2023-08-01

**Authors:** Fany Blanc, Nicolas Bertho, Guillaume Piton, Jean-Jacques Leplat, Giorgia Egidy, Emmanuelle Bourneuf, Silvia Vincent-Naulleau, Armelle Prévost-Blondel

**Affiliations:** 1 INSERM, U1016, Institut Cochin, 75014 Paris, France; 2 Université Paris-Saclay, INRAE, AgroParisTech, GABI, 78350 Jouy-en-Josas, France; 3 CEA, DSV/iRCM/SREIT/LREG, 78350 Jouy-en-Josas, France; 4 Université Paris‐Saclay, INRAE, VIM, 78350 Jouy-en-Josas, France; 5 INRAE, Oniris, BIOEPAR, 44300 Nantes, France; 6 Laboratoire de Cancérologie Expérimentale, CEA/DRF/IBFJ/IRCM, 92265 Fontenay-Aux-Roses, France; 7 Université Paris Cité, Paris, France; 8 Plateforme animalerie, CEA/DRF/IBFJ/IRCM, 92265 Fontenay-Aux-Roses, France; 9 Bureau des Etudes Biomédicales chez l’Animal, CEA/DRF/BEBA, 92265 Fontenay-Aux-Roses, France; 10 CNRS, UMR8104, Paris, France

**Keywords:** Tumor immune microenvironment, Tumor-associated macrophages, Spontaneous tumor regression, Melanoma

## Abstract

The human cutaneous metastatic melanoma is the deadliest skin cancer. Partial, or less frequently complete spontaneous regressions could be observed, mainly mediated by T cells. Nevertheless, the underlying mechanisms are not fully unraveled. We investigated the first events of the immune response related to cancer regression in Melanoma-bearing Libechov Minipigs (MeLiM), a unique swine model of cutaneous melanoma that regresses spontaneously. Using a multiparameter flow cytometry strategy and integrating new clinical and histological criteria of the regression, we show that T cells and B cells are present only in the late stages, arguing against their role in the initial destruction of malignant cells. NK cells infiltrate the tumors before T cells and therefore might be involved in the induction of the regression process. Myeloid cells represent the main immune population within the tumor microenvironment regardless of the regression stage. Among those, MHCII^+^ CD163^−^ macrophages that differ phenotypically and functionally compared to other tumor-associated macrophages, increase in number together with the first signs of regression suggesting their crucial contribution to initiating the regression process. Our study supports the importance of macrophage reprogramming in humans to improve current immunotherapy for metastatic melanoma.

**Supplementary Information:**

The online version contains supplementary material available at 10.1007/s00262-023-03503-6.

## Introduction

The cutaneous melanoma is the deadliest skin cancer. Its incidence has steadily increased since the 1970s with a predominance in fair-skinned populations from Australia, New Zealand, North America and northern Europe [1]. The high mortality rate caused by the disease is due to its high rate of metastasis. Nevertheless, spontaneous regressions, partial or less frequently complete, are observed macroscopically and histologically with an overall incidence ranging from 10 to 35% and even reaching 58% in primary melanoma with a Breslow thickness below 1 mm [2, 3]. The progressive disappearance of malignant cells is associated with fibrosis, presence of lymphocytes and melanophages and varying degrees of neovascularization, depigmentation around the tumors, and inflammation. Although the mechanisms of spontaneous regression are not fully unraveled, the host immune system plays a key role, attested to by the early activation of immunity against primary cutaneous melanoma leading to lymphocytic and histiocytic infiltration in regressing lesions [4, 5]. The immune reactions toward “self” cancer antigens may lead to the destruction of normal melanocytes and subsequently depigmentation occasionally occurring in patients. Such auto-immunity has even been associated with a favorable melanoma prognosis [3, 6, 7].

In-depth understanding of new immune parameters could contribute to guide the development of novel cancer treatments [8, 9]. In particular, the identification of immune cells related to the spontaneous tumor regression is of great interest but remains difficult in humans due to its low prevalence and the limited access to lesions with active regression. In addition, the extent of cancer regression is not objectively defined by a universal scheme, leading to an unreliable reporting of regression that may explain the conflicting data regarding on prognostic importance of regression [10]. Melanoma-bearing Libechov Minipigs (MeLiM) spontaneously develop multifocal primary cutaneous melanomas around birth, with clinical and histopathological features comparable to human counterparts [11, 12]. 80% of animals exhibit multiple lesions from benign to highly invasive, eventually leading to lymph nodes and visceral metastasis. A regression occurs without any treatment 2 to 4 months after birth, corresponding to a complete disappearance of primary tumors and metastases. Overall, only a 4% mortality rate is observed, likely due to metastatic complications appearing before the regression onset. Our team has already described clinically and histologically the regression process often accompanied by a local or systemic depigmentation of hair, skin and eyes [12, 13] and reported its association with the modulation of immune-related genes with humoral, monocyte/macrophage-like, and T/NK signatures [14]. In the MeLiM model, we have recently evidenced changes in the circulating immune cell composition due to melanoma occurrence: young melanoma-bearing piglets harboring higher proportions of NK cells, CD4^+^ and CD4^+^ CD8α^+^ T cells, and CD21^−^ B cells among B cells consistent with the immune-mediated spontaneous regression [15]. Others have shown the involvement of fibronectin and tenascin C in forming fibrous tissue during the spontaneous tumor regression [16].

Here, we performed a longitudinal analysis of melanoma lesions from a large cohort of MeLiM pigs, from the initiation to latest stages of tumor regression. Using our recent multiparametric flow cytometry strategy [15] and integrating new clinical and histological criteria of the regression, we studied the tumor microenvironment and identified the different subsets of T, B, NK cells and myeloid cells infiltrating melanoma lesions. We also evidenced a macrophage subset exhibiting very different functions compared to other tumor-associated macrophages (TAMs) and that increases with the first signs of regression, supporting their contribution to the initiation of the regression process.

## Methods

### Experimental pigs

MeLiM pigs (14 males and 20 females) from 13 different litters were examined for the presence of cutaneous melanoma every 1 to 2 weeks until 3 months of age and then every 2 to 3 weeks until 5 months. All animals were genotyped for a specific haplotype segregating in MeLiM, responsible for epitope deficiency to the anti-CD4 antibody [13]. Clinical examination consisted in identifying new pigmented melanocytic lesions, monitoring the evolution of previously identified lesions and examining the presence of palpable lymphadenopathies (supplementary Tables 1 and 2). Some tumors were excised surgically under general anesthesia and either stored in liquid nitrogen for protein extraction, in 10% buffered formalin for histological analyses, or in PBS containing 5% of Vetedine solution (Vetoquinol), 200 units/mL of penicillin, 200 µg/mL of streptomycin and 0.2% of Fungizone (all from Gibco) for further processing of enzymatic digestion.

### Histopathology

Hematoxylin–eosin-saffron—stained paraffin-embedded sections were evaluated histologically according to the human classification. Regression was characterized histologically by the presence of a zone of dermal fibrosis and if so, other criteria were examined in the regressive zone (supplementary Table 2).

### Immunohistochemical staining and image analysis

Immunohistochemical stains for CD3 (mouse IgG1 anti-pig CD3, clone PPT3, 2.5 µg/mL, SouthernBiotech) were performed on 5-μm thick paraffin-embedded tissue sections using standard techniques after unmasking at 90 °C for 1h, ImmPRESS^®^ HRP Anti-Mouse IgG (Peroxidase) kit (LSBio) and HRP green and PAS hematoxylin counterstain. Images were acquired with the Pannoramic SCAN digital slide scanner (objective magnification X40). Zones were defined manually and CD3 expression was quantified in each zone using with the CaseViewer and QuantCenter softwares (all from 3DHISTECH) and expressed as HRP green colored pixels relatively to 1000 total pixels. Partition of connective tissue vs tumoral cells was performed on Trichrome- or HES-stained sections using the same softwares.

### Tumoral protein extraction and cytokine assays

Proteins were extracted from frozen tumors in protein lysis buffer (20mM Tris HCl pH7.5, 150 mM NaCl, 1mM EDTA, 1% NP-40, 0.1% SDS, 0.5% sodium deoxycholate) containing protease and phosphatase inhibitor (Pierce). Protein concentrations were measured with the BCA protein assay kit (Pierce) and cytokines were assessed on 12.5 µg of total protein. IFNα, IFNγ, IL-2, IL-4, IL-6, IL-8, IL-10, IL-17, IL-1β and TNFα were measured by a Cytokine Bead Assay as previously described [17]. CCL2 and TGFβ were assessed using Swine CCL2 VetSet ELISA Development Kit (KingFisher Biotech) and Mouse/Rat/Porcine/Canine TGF-beta 1 Quantikine ELISA Kit (R&D systems) following the manufacturers’ instructions (supplementary Table 3).

### Enzymatic digestion of lesions and identification of cells

Epidermis and hypodermis were carefully removed and tumor tissues were weighted. Single cell suspensions were obtained by an enzymatic treatment for 1 h at 37 °C under agitation with collagenase B at 4 mg/mL and DNase I at 0.1 mg/mL (Roche) in DMEM containing 100 units/mL penicillin, 100 µg/mL streptomycin, 2 mM l-Glutamine, 0.5 mM EDTA and 2% FBS (all from Gibco). Extensive washings were performed in the same medium without enzymes to remove released melanin.

Absolute number and viability of cells were determined with the ViaCount Assay performed on easyCyte 6HT-2L Guava flow cytometer (Millipore) following manufacturer’s instructions. Cells were stained as previously described for blood cell analysis [15]. Briefly, three combinations of antibodies, listed in supplementary Table 4, with the aqua LIVE/DEAD^®^ Fixable Dead Cell Stain Kit (Thermo Fisher Scientific) for viability, were used to identify lymphoid cells (combinations A: CD45, CD3, CD8α, CD4, γδTCR and CD16 and B: CD45, MHC II, CD21 and CD79a) and myeloid cells (combination C: CD45, MHC II, PG68A, CD163, CD172a, CD14 and CADM1). Five million cells were processed in each combination and finally fixed in BD CellFIX solution before analysis on a BD LSR Fortessa cytometer (BD Biosciences). Data were analyzed using FlowJo V10 software (supplementary Table 5).

### T and NK cell functionality

After enzymatic digestion of tumoral lesions, five million cells were incubated in 1 mL of RPMI medium containing 5% FBS, 100 units/mL penicillin, 100 µg/mL streptomycin, 2 mM l-Glutamine, 10 mM Hepes (all from Gibco) with or without PMA (50 ng/mL, from Sigma-Aldrich) and ionomycin (1 µg/mL, from Sigma-Aldrich) for 3 h at 37 °C in the presence of monensin (1/1000, eBioscience) and brefeldin A (1/1000, Invitrogen). Cells were then stained for viability with the Near IR LIVE/DEAD^®^ Fixable Dead Cell Stain Kit (Thermo Fisher Scientific) and stained with a combination of antibodies targeting CD45, CD3, CD8α and CD16 (combination D in supplementary Table 4). Detection of IFNγ was done by intracellular staining using Foxp3/Transcription Factor Staining Buffer Set (eBioscience) as previously described [15]. IFNγ intracellular staining specificity was determined by incubating cells with labeled isotype control. Cells were then fixed in BD CellFIX solution before analysis on a BD LSR Fortessa cytometer (BD Biosciences). The data analysis was performed using FlowJo V10 software.

### Macrophage subpopulation cell sorting and qRT-PCR

After enzymatic digestion of tumoral lesions, cells were stained with the combination for myeloid cells (combination C in supplementary Table 4: CD45, MHC II, PG68A, CD163, CD172a, CD14 and CADM1) but not fixed. Macrophages subpopulations were sorted using a FACS Aria III cell sorter (BD Biosciences; 100 µm nozzle). RNA from sorted cells were extracted using the Arcturus PicoPure RNA Isolation kit (ThermoFisher Scientific) according to manufacturer’s instructions. Their concentrations were measured with a NanoDrop 2000 spectrophotometer (16.9 ± 13.8 µg were obtained per sample) and RNA integrity was assessed by an Agilent 2100 Bioanalyzer using Eukaryote total RNA 6000 Nano Kit (RIN obtained were 8.7 ± 0.6, ranging from 7.2 to 9.7). RT-PCR was performed from 10 to 100 ng of RNA using TaqMan Reverse Transcription Reagents (Applied Biosystems, ThermoFisher Scientific). Relative mRNA expression was evaluated by qPCR using either the Fast SYBR™ Green Master Mix or TaqMan Fast Advanced Master Mix on the QuantStudio 12K Flex system (all from Applied Biosystems) with the primers described in supplementary Table 6. A Ct value equal to 40 was set for non-detected genes. Gene expressions were normalized using the ΔCt method by subtracting the Ct obtained for the gene of interest by the geometric mean of the Ct obtained for Ribosomal protein S24 (*RPS24*) and Ribosomal protein L32 (*RPL32*) reference genes. Relative gene expression (RQ) were calculated by subtracting the ΔCt(sample) from the ΔCt(mean) across all samples and the 2^−ΔΔCt^ method.

### Statistics

Data analyses were performed using R (v3.6.1). For statistical inference, different transformations have been applied on data: a square root transformation for percentages of connective tissue, a log base 10 + 1 transformation for cytokine concentration and a log base 2 transformation for qPCR relative gene expression. Factorial Analysis of Mixed Data (FAMD) and Principal Component Analysis (PCA) were performed using FactoMineR (v2.1) package and visualized with the factoextra R package (v1.0.6). Statistical description of groups by the quantitative and qualitative variables were performed using *v* test obtained using the catdes function. Eventually, the imputePCA function of missMDA (v1.16) package was used to handle missing values. Heatmaps were built using ComplexHeatmap (v2.0.0) package.

Effect of sex and regression stages was evaluated on the different proportions of cell populations among immune cells in the tumors using a linear mixed model using the lm function with sex (two levels) and regression stages (5 levels) as fixed effects. *p* values for fixed effects were obtained using the lmerTest package (v3.1-1) by type III ANOVA tables with Satterthwaite’s approximation to degrees of freedom using the ANOVA function. Sex effect was not significant. Pairwise comparisons with Tukey’s adjustment were performed to assess the differences within regression stages using the emmeans package (v1.5.2-1). A significance level of 0.05 was applied. All significant F and *p*-values are reported in the supplementary Tables 7–9 as mentioned in figure legends. Results in the text are expressed as mean ± SD. Other statistical tests used are mentioned in the appropriate figure legend.

## Results

### Clinical and histological features of spontaneous melanoma regression in MeLiM pigs

**Fig. 1:**
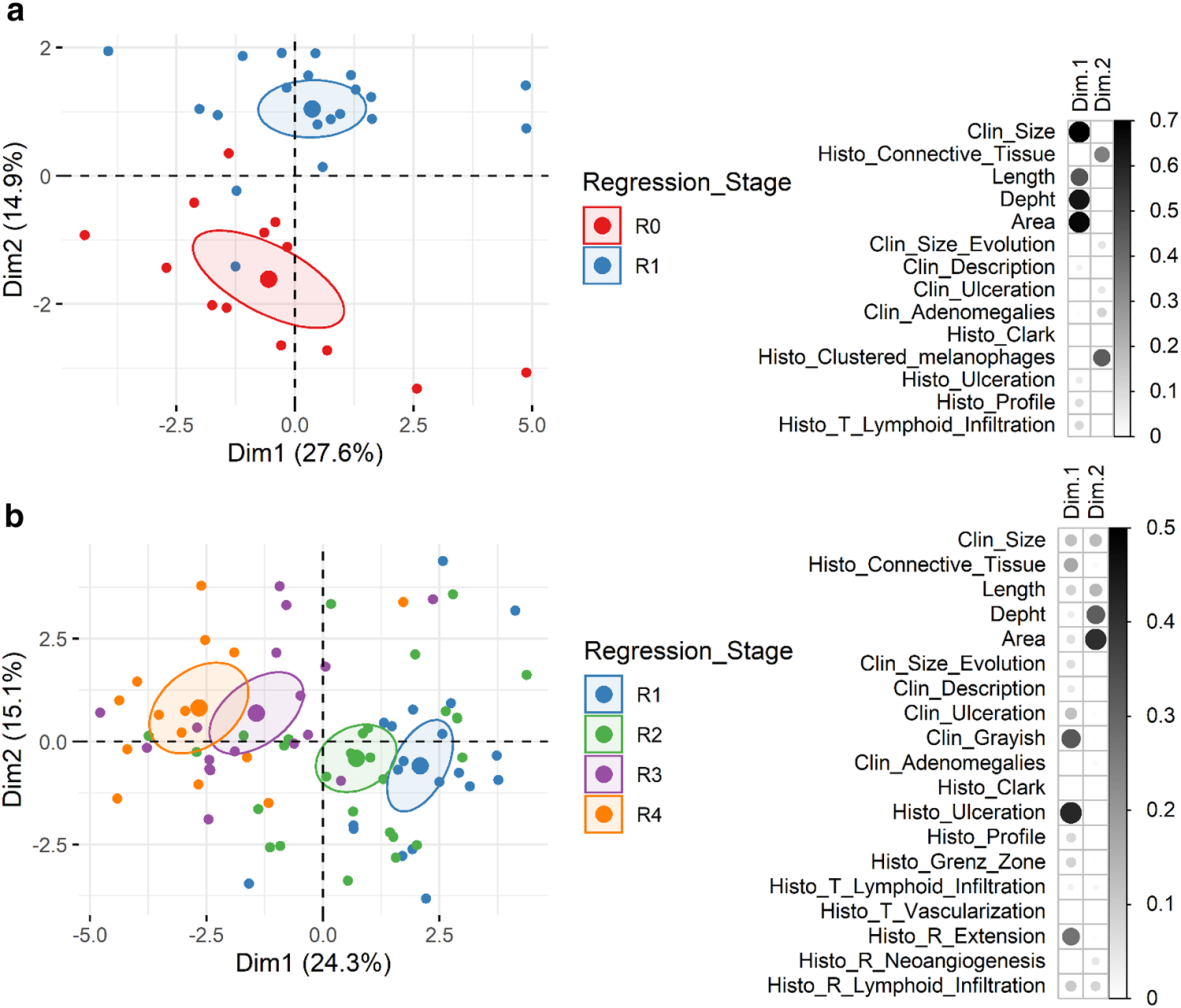
Factorial Analysis of Mixed Data (FAMD) of melanoma lesions with clinical and histological data. **a** Lesions sampled between 14 and 49 days without (R0) or with partial regression (R1). **b** Lesions in regression from groups R1 to R4: R1 from 19 to 49 days, R2 from 51 to 69 days, R3 from 72 to 94 days and R4 from 100 to 154 days. On the left, individuals are represented in the first 2 dimensions of the FAMD and colored by regression stages. On the right, cos2 values represent the quality of representation of the different qualitative and quantitative variables on the factor map reported for the first 2 dimensions

A total of 92 cutaneous pigmented lesions from 34 pigs aged 14 to 154 days were analyzed. Supplementary Tables 1 and 2 present the pig’s individual clinical data with the histological and clinical parameters, respectively. Lesions corresponded to invasive nodular melanoma equivalent to Clark’s level IV or V. The tumor area consisted of highly pigmented cells. Regression was characterized histologically by the presence of a zone of dermal fibrosis. Melanoma without any microscopic regression, collected from animals aged 14 to 48 days, were classified as R0. Melanoma from age-matched animals with a partial zone of regression were classified as R1. The stage of regression was then determined kinetically: R2 from 51 to 69 days, R3 from 72 to 94 days and R4 from 100 to 154 days. Based on the melanoma clinical data and on the histological features of tumoral and regressive zones (Table 1), FAMD allowed to identify many characteristics of progressive R0 lesions and R1 lesions in partial regression (Fig. 1a). Indeed, in animals belonging to the group R1, lesions size levels off, adenomegalies are more frequent, and lesions display more connective tissue and clustered melanophages corresponding to dense nests of heavily pigmented melanin-laden histiocytes. Both R0 and R1 lesions are frequently ulcerated but R0 lesions more intensely.

**Table 1:** Clinical and histological features of spontaneous melanoma regression in MeLiM pigs

Stages of regression	R0 (*n* = 13)	R1 (*n* = 20)	R2 (*n* = 28)	R3 (*n* = 17)	R4 (*n* = 14)	Link between stages of regression and variables (*p* value)	
						R0 vs R1	R1 to R4
Clinical observations of the animal and lesions							
Lesion size (mean in cm)	2.80	3.03	2.90	2.55	**2.33**		3.38E−02
Lesion size’s evolution						4.17E−02	1.40E−03
Increasing (%)	**92.3**	**60.0**	39.3	**29.4**	14.3		
Stable (%)	**7.7**	**40.0**	60.7	76.5	64.3		
Decreasing (%)	0.0	0.0	0.0	5.9	21.4		
Description							4.38E−02
Plateau (%)	46.2	**25.0**	67.9	58.8	78.6		
Dome (%)	53.8	60.0	25.0	35.3	21.4		
Exophytic (%)	0.0	15.0	7.1	5.9	0.0		
Ulceration							8.06E−04
No (%)	30.8	35.0	60.7	**94.1**	**100**		
Yes (+) (%)	44.4	76.9	81.8	**100**			
Yes (++) (%)	55.6	23.1	18.2	0			
Grayish lesion (%)	0	**5.0**	**25.0**	**76.5**	**92.9**		3.70E−08
Palpable adenomegalies (%)	**38.5**	85.0	89.3	100	100	5.59E−03	
Histological observations of the tumoral zone							
Profile							4.64E−04
Dome or plateau (%)	66.7	**40.0**	71.4	88.2	**100**		
Polypoid (%)	33.3	**60.0**	28.6	11.8	**0**		
Size of the lesion’s section							
Length (mean in cm)	2.17	1.98	1.98	1.84	1.70		
Depht (mean in cm)	0.84	0.98	0.93	0.93	0.92		
Area (mean in mm^2^)	156.2	149.2	141.2	143.2	130.9		
Clark’s level							
IV (%)	8.3	**15.8**	44.4	47.1	50.0		
V (%)	91.7	**84.2**	55.6	41.2	50.0		
Presence of Grenz zone (%)	0	**0**	**3.6**	6.3	**71.4**		3.95E−09
Connective tissue (mean of %)	**6.6**	**16.7**	**19.0**	**27.6**	**34.4**	1.48E−04	9.89E−07
Ulceration (%)	92	**95.0**	71.4	**35.3**	**28.6**		6.24E−05
High vascularization (%)	100	100	92.9	100	100		
Clusters of melanophages (%)	**0**	100	100	100	100	9.22E−09	
Lymphoid infiltration							6.59E−04
No (%)	76.9	**65.0**	46.4	25.0	**0**		
Yes (perilesional) (%)	66.7	57.1	53.3	16.7	**53.8**		
Yes (intralesional) (%)	33.3	**42.9**	46.7	**83.3**	46.2		
Histological observations of the regressive zone							
Extension							1.11E−06
Dermal (%)	–	**100**	**85.2**	50.0	**21.4**		
Dermal and epidermal (%)	–	**0**	**14.8**	50.0	**78.6**		
Neoangiogenesis (%)	–	70.0	85.7	94.1	92.9		
Lymphoid Infiltration (%)	–	**20.0**	**32.1**	**94.1**	**85.7**		8.18E−07

Melanoma lesions were assigned to different stages: lesions sampled between 14 and 49 days without (R0) or with partial regression (R1); and older lesions in regression from groups R2 to R4, determined kinetically (R2 from 51 to 69 days, R3 from 72 to 94 days and R4 from 100 to 154 days). In the table, the proportion of lesions with the specific phenotype is indicated for each stage of regression for the qualitative variables; mean is indicated for the quantitative variables. *p* values obtained after a FAMD analyses for the link between stages of regression and variables are also indicated when significant. Values are in bold when they are statistically different for this stage of regression comparing to others.

Separation of lesions in regression from R1 to R4 groups is more continuous (Fig. 1b and Table 1). While the regression occurs, lesion size stabilizes and then decreases, Clark’s level and ulceration reduce, and tumors become grayish. Histologically, progressively with age, cutaneous melanoma becomes flat and the tumoral zone decreases as the lymphoid infiltration and proportion of connective tissue increase. A grenz zone could be observed at the last time-point. Regression first occurs partially in the dermis and then extends to the epidermis. Lymphoid infiltration is more frequent in R3 and R4 stages of regression.

### Evolution of cytokine environment in melanoma lesions along the regression process

**Fig. 2:**
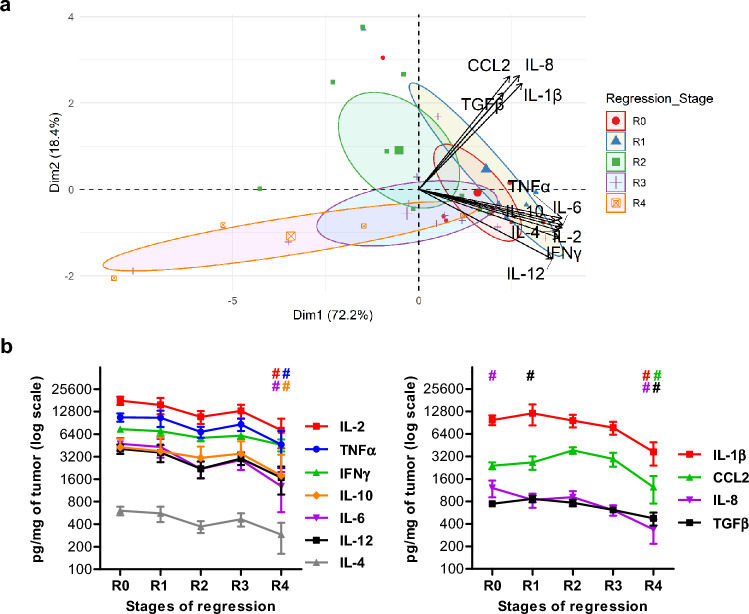
Cytokine profile in melanoma lesions along the regression process. **a** Biplot of individuals and variables after a Principal Component Analysis (PCA) of cytokine concentrations assessed in tumors. Each melanoma lesion is represented in the first two dimensions of the PCA and colored according to its stage of regression. Confidence concentration ellipses are represented for each group (confidence level set to 95%). **b** Representation of cytokine concentrations assessed in the tumors in the two clusters defined by the PCA. (*n* = 8 for R0, *n* = 6 for R1, *n* = 8 for R2, *n* = 10 for R3 and *n* = 4 for R4). Statistical analysis was performed using v test after PCA analysis, a significant *p* value (< 0.05) is represented by a # which color corresponds to the appropriate cytokine when different from others regression stages. All significant *v* tests and *p* values are reported in supplementary table 7

Thirteen cytokines have been measured in tumoral lesions along the regression process (supplementary Table 3). IL-17 and IFNα, weakly expressed (52.3 and 214.4 pg/mg of tumor, respectively), have not been included in further analyses. IL-4, TGFβ and IL-8 were assessed in low amounts (471 to 820 pg/mg of tumor); CCL2, IL-12, IL-6 and IL-10 in moderate amounts (2825 to 3440 pg/mg of tumor); and IFNγ, TNFα, IL-1β and IL-2 in high amounts (6293 to 13,492 pg/mg of tumor). Cytokine environment in tumors has then been studied by a multiple factor analysis. The first two principal components after a PCA explain 90.6% of the variation within the dataset. The plot in Fig. 2a illustrates the relative contribution of each cytokine to the two principal components and highlights two clusters of cytokines. IL-2, TNFα, IFNγ, IL-6, IL-10, IL-12 and IL-4 correlate together and are main contributors to the first dimension. IL-1β, CCL2, IL-8 and TGFβ are more spread along the second-dimension axis. The evolution profile of these clusters along the regression is represented Fig. 2b. Tumors with no regression (R0 group) express more IL-8 and tumors initiating regression (R1) express more TGFβ. On the opposite, less IL-2, TNFα, IL-6, IL-1β, IL-10, CCL2, IL-8 and TGFβ are found at the latest stage of regression (R4). Thus, the cytokine profile in tumors evolves along the regression process, with a significant decrease for nearly all the cytokines measured.

### Phenotypic characterization of melanoma-infiltrating immune cells during the regression process

**Fig. 3:**
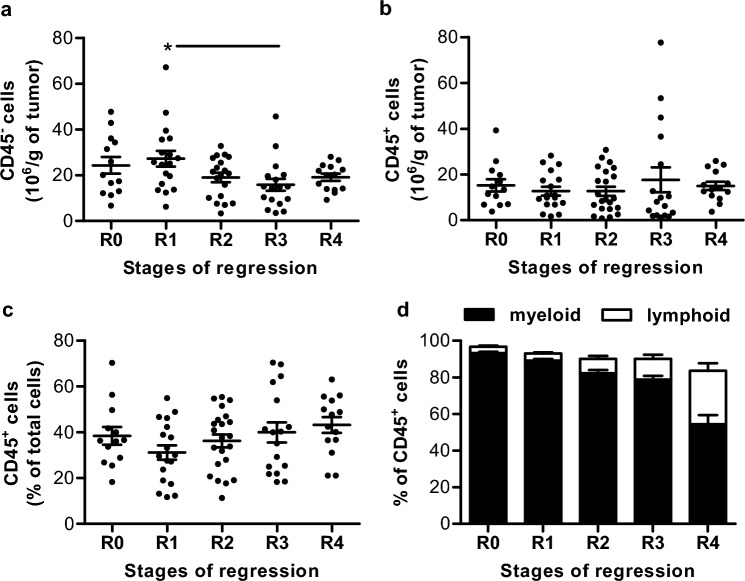
Immune cells infiltrate melanoma lesions during the regression process. **a, b** Number of non-immune CD45^−^ (**a**) and immune CD45^+^ (**b**) cells per gram of tumor. **c** Proportion of CD45^+^ cells among total cells. **d** Proportion of myeloid (CD172a^+^) and lymphoid (CD3^+^ or CD79a^+^ or CD3^−^ CD8^+^ CD16^+^) cells among immune cells. In the plots, bars represent the means ± SEM; (*n* = 13 for R0, *n* = 18 for R1, *n* = 22 for R2, *n* = 17 for R3 and *n* = 14 for R4). Statistical analysis: a significant *p* value (< 0.05) is represented by a * and *F* and *p* values are reported in supplementary Table 8

Immune cells infiltrating melanoma lesions have been analyzed by multicolor flow cytometry using the gating strategy we recently published [15]. All individual data are reported in supplementary Table 5. The number of non-immune cells (CD45^−^) per gram of tumor tends to decrease along the regression, from 27.2 million on average at R1 to 15.8 (*p* = 0.023) and 19.1 million at R3 and R4, respectively (Fig. 3a). The number of immune cells (CD45^+^) is quite stable with a mean of 14.5 million of CD45^+^ cells extracted per gram of tumor (Fig. 3b). They represent 38.4% of total cells (ranging from 18.2 to 70.2%) and slightly increase between R1 and R4 stages (Fig. 3c). Interestingly, the proportions of immune cell subtypes differ during the regression process (Fig. 3d). Myeloid cells account for 93.3% of immune cells associated with the tumor in R0 lesions, but only 54.4% in R4 lesions. In contrast, lymphoid cells represent only 3.4% of cells in R0 lesions, but reach respectively 11.4% and even 29.1% of them at R3 and R4 stages. Immune cell subtypes that have evolved during the regression process are detailed below.

### Lymphoid cells infiltrate the tumor at the latest stage of regression

**Fig. 4:**
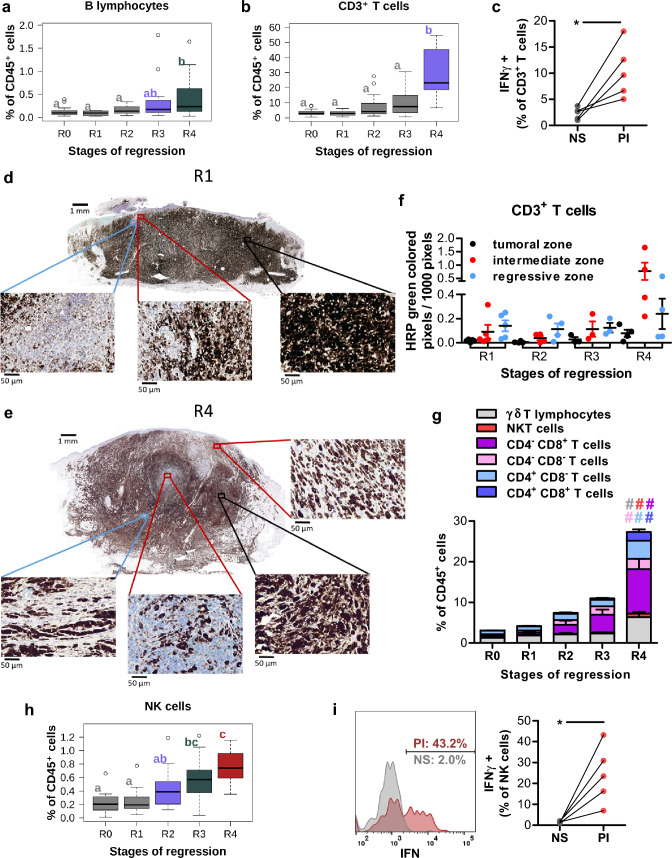
Lymphoid cells infiltrate melanoma at the latest stage of regression. **a** B lymphocytes (CD79a^+^) proportion among tumor-infiltrating immune cells. Letters and colors correspond to statistically different groups (*n* = 12 for R0, *n* = 12 for R1, *n* = 19 for R2, *n* = 17 for R3 and *n* = 14 for R4, see supplementary Table 8 for exact *F* and *p* values). **b** Proportion of CD3^+^ T cells among tumor infiltrating CD45^+^ cells during the different regression stages. Letters and colors correspond to statistically different groups (*n* = 13 for R0, *n* = 17 for R1, *n* = 20 for R2, *n* = 17 for R3 and *n* = 14 for R4, see supplementary Table 8 for exact *F* and *p* values). **c** IFN-γ secretion by CD3^+^ T cells gated from a single cell suspension, derived from 5 tumors belonging to the R2 stage of regression, either not stimulated (NS) or after stimulation with PMA/Ionomycin (PI). Statistical analysis: paired *t* test (*p < 0.05). **d, e** In situ staining of CD3^+^ T cells (HRP green with PAS hematoxylin counterstain) of melanoma lesions at an early (**d**) or late (**e**) stage of regression. **f** Quantification of the CD3 HRP green staining in tumoral, intermediate and regressive zones in tumors. **g** Percentages of the different T cells subsets γδ T lymphocytes, NKT cells, CD4^−^ CD8α^+^, CD4^−^ CD8α^−^, CD4^+^ CD8α^−^ and CD4^+^ CD8α^+^ T lymphocytes within tumor-infiltrating CD45^+^ cells (*n* = 12 for R0, *n* = 12 for R1, *n* = 19 for R2, *n* = 17 for R3 and *n* = 14 for R4, a significant *p* value (< 0.05) is represented by a # which color corresponds to the appropriate cell population, see supplementary Table 8 for exact *F* and *p*-values). **h** Proportion of NK (CD3^−^ CD8a^+^ CD16^+^) cells among tumor-infiltrating CD45^+^ cells during the different regression stages. Letters and colors correspond to statistically different groups (*n* = 13 for R0, *n* = 18 for R1, *n* = 20 for R2, *n* = 17 for R3 and *n* = 14 for R4, see supplementary Table 8 for exact *F* and *p* values). **i** IFN-γ secretion by NK cells gated from a single cell suspension, derived from 5 tumors belonging to the R2 stage of regression, either not stimulated (NS) or after stimulation with PMA/Ionomycin (PI). One representative histogram plot (left panel) and % of IFNγ^+^ NK cells (right panel). Statistical analysis: paired *t* test (**p < 0.01)

B cells poorly infiltrate tumors, and reach 0.47% of immune cells at stage 4 of regression (Fig. 4a). CD3^+^ T cells are present in few amounts at the beginning of regression (R1 to R3) and only in a high proportion (27.9% of CD45^+^ cells) at stage 4 (Fig. 4b). CD3^+^ T cell functionality of R2 stage lesions has been evaluated by measuring their secretion of IFN-γ by intracellular staining following stimulation of single cell suspension of tumor cells with PMA/Ionomycin (Fig. 4c) and 10.4% of them (range 5–18%) produce IFN-γ after stimulation *vs* 2.3% when non-stimulated. Immunohistochemistry reveals that CD3^+^ T cells are located in regressive and intermediate zones from the beginning of the regression and particularly highly concentrated at stage 4. They were also present in remaining tumor zones only at the latest stage of regression (Fig. 4d–f). All T cell subtypes (CD4^−^ CD8α^+^, CD4^+^ CD8α^−^, CD4^+^ CD8α^+^, CD4^−^ CD8α^−^, γδ and NKT T cells) exhibit the same evolution profile along regression, with a statistically significant presence in R4 lesions (Fig. 4g). The majority of them at the latest stage of regression are CD4^−^ CD8α^+^ T cells representing on average 11.0% of immune cells, followed by γδ T cells (6.4% of immune cells), CD4^+^ CD8α^−^ T cells (4.5% of immune cells), CD4^−^ CD8α^−^ T cells (3.1% of immune cells), CD4^+^ CD8α^+^ T cells (2.1% of immune cells) and NKT cells (0.82% of immune cells). Indeed, in the MeLiM model, B and T cells are detected within melanoma lesions only at the latest stage of regression and may thus be the consequence of this process.

NK cells (CD45^+^ CD3^−^ CD8α^+^ CD16^+^ cells) represent 0.17 and 0.27% of immune cells in tumor lesions of pigs at R0 and R1 stages, respectively. This proportion increases regularly along the regression process reaching 0.59 and 0.75% at R3 and R4 stages (Fig. 4h). NK cell functionality of R2 stage lesions has been evaluated by measuring their secretion of IFN-γ by intracellular staining following stimulation of single cell suspension of tumor cells with PMA/Ionomycin (Fig. 4i). Interestingly, 30.0% of NK cells (range 11.4–42.3%) produce IFN-γ after stimulation, supporting their putative involvement in the tumor regression in vivo.

### Slight modulation of PMN and DC during the course of regression

**Fig. 5:**
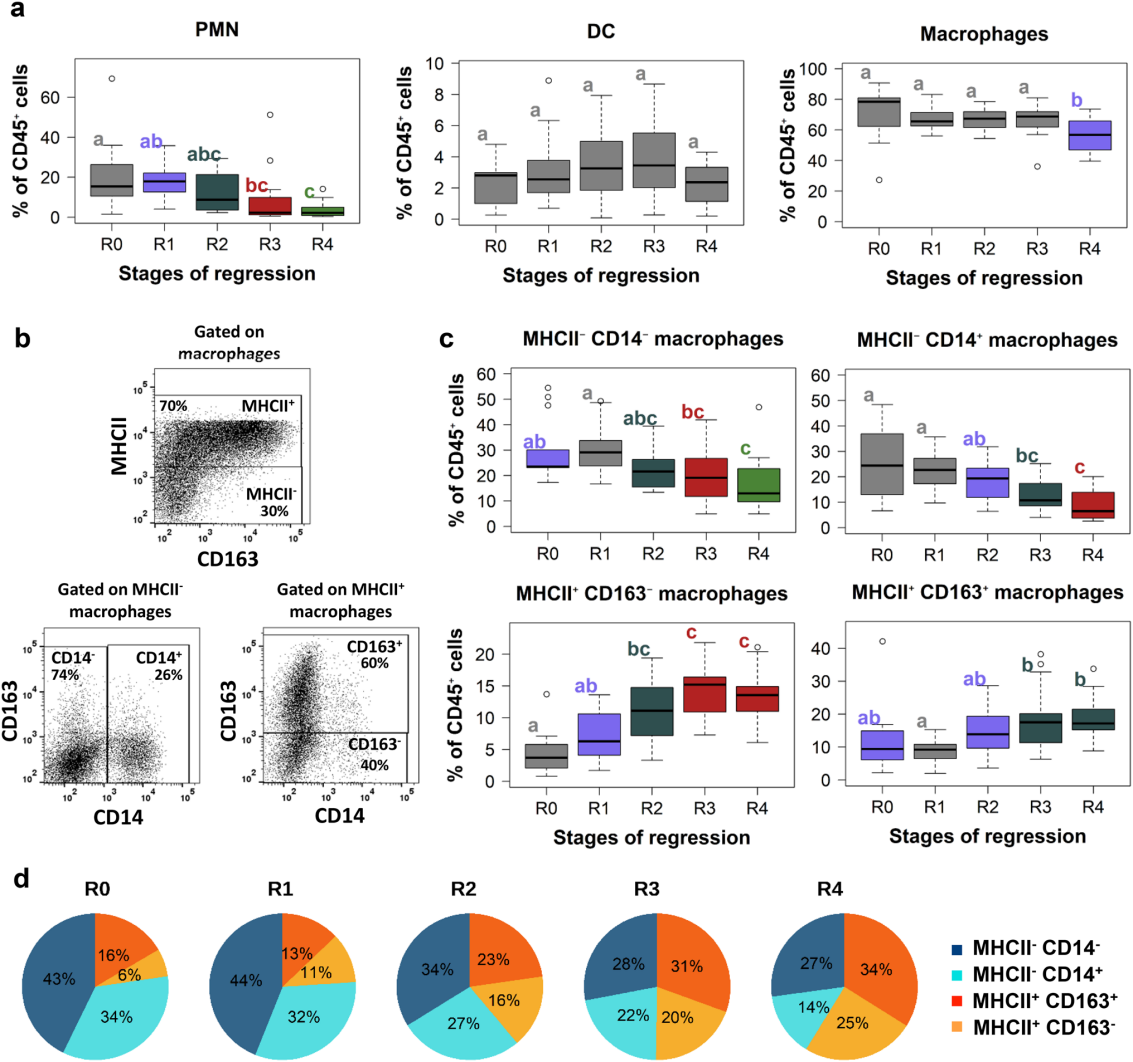
Modulation of myeloid cells during the regression: MHCII^+^ TAMs accumulate from the earliest stage of the melanoma regression. **a** Proportion of PMN (CD172a^+^ PG68A^+^), DC (CD172a^+^ MHCII^hi^ FSC^hi^) and TAMs (CD172a^+^ PG68A^−^ MHCII^−/low^) among tumor-infiltrating CD45^+^ cells during the different regression stages. Letters and colors correspond to statistically different groups (*n* = 13 for R0, *n* = 17 for R1, *n* = 20 for R2, *n* = 17 for R3 and *n* = 14 for R4, see supplementary Table 8 for exact *F* and *p* values). **b** Gating strategy to identify different subsets of TAMs. After exclusion of doublets, live/CD45^+^/CD172a^+^/non DC/non PMN cells were represented in a CD163 vs MHCII dot plot. MHCII^−^ and MHCII^+^ TAMs were evaluated in CD14 vs CD163 dot plots. Four major subsets of TAMs were identified: MHCII^−^ CD14^−^, MHCII^−^ CD14^+^ (lower left panel); MHCII^+^ CD163^−^ and MHCII^+^ CD163^+^ (lower right panel). **c** Percentages of the four TAMs subsets among tumor-infiltrating CD45^+^ cells during the different regression stages (*n* = 13 for R0, *n* = 17 for R1, *n* = 15 for R2, *n* = 17 for R3 and *n* = 14 for R4, see supplementary Table 8 for exact *F* and *p* values). **d** Pie charts representing the proportion of the four different subsets among TAMs at the different regression stages

Despite the decrease in proportion of myeloid cells among tumor-infiltrating immune cells in the course of the regression process, myeloid cells represent the main population within the tumor microenvironment whatever the regression stage (Fig. 3d). Among those, polymorphonuclear (PMN) cells represent 20.0% of cells before regression, and decrease all along the regression process reaching 8.2 and 3.9% at R3 and R4 stages, respectively (Fig. 5a). DC remained at low levels in the tumors (ranging from 2.2 to 3.9% of immune cells) without statistically significant evolution during regression (Fig. 5a). All subtypes of DC we have investigated evolve similarly (supplementary Fig. 1).

### MHCII^+^ TAMs accumulate from the beginning of the regression process

TAMs proportion remains stable from R0 to R3 (ranging from 66.5 to 71.0% on average among CD45^+^ cells) and decreased at the R4 stage where they represent 55.9% of immune cells (Fig. 5a). Four major subsets of TAMs were identified considering their expression of MHCII, CD163 and CD14: MHCII^−^ CD14^−^, MHCII^−^ CD14^+^, MHCII^+^ CD163^−^ and MHCII^+^ CD163^+^ (Fig. 5b). MHCII^−^ TAMs (CD14^−^ or CD14^+^) express low levels of CD163 and MHCII^+^ TAMs (CD163^−^ or CD163^+^) low levels of CD14. Interestingly, as illustrated in Fig. 5c, the proportion of the different subtypes among immune cells evolves along the regression. Indeed, MHCII^−^ CD14^−^ and MHCII^−^ CD14^+^ TAMs significantly decrease with regression, particularly at R3 stage and at R4 stage, respectively. On the opposite, the proportion of MHCII^+^ TAMs among immune cells increases with regression. Interestingly, that of MHCII^+^ CD163^−^ TAMs doubles between R0 and R1 lesions derived from age-matched animals, at the earliest step of the regression, and significantly increases at R2 stage (Fig. 5C). When representing the four TAMs subsets in percentages of all TAMs (Fig. 5d), their phenotypes evolve in huge proportion: MHCII^−^ TAMs decrease by half, representing 43 and 34% of total macrophages at R0 for CD14^−^ and CD14^+^, respectively, and 27 and 14% of them at the end of regression. Conversely, MHCII^+^ TAMs increase, from 16 to 34% for CD163^+^ and from 6 to 25% for CD163^−^ when comparing R0 and R4 stages.

### CD163^−^ MHCII^+^ TAMs are functionally very different from other TAMs subsets

**Fig. 6:**
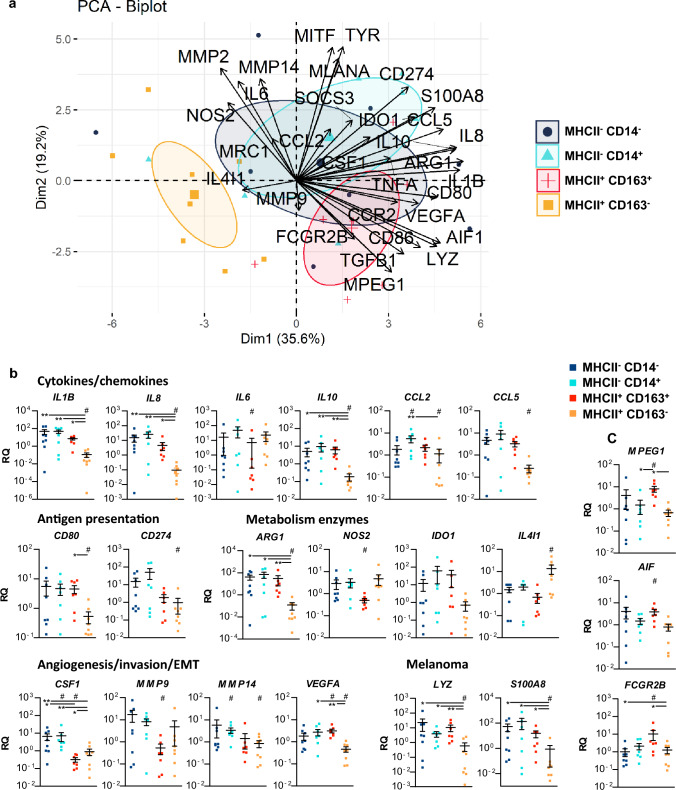
Gene expression analysis TAMs subsets: MHCII^−^ CD14^−^, MHCII^−^ CD14^+^, MHCII^+^ CD163^−^, and MHCII^+^ CD163^+^. **a** Biplot of individuals and variables after a Principal Component Analysis (PCA) of relative gene expression in subsets of macrophages obtained after cell sorting for 8 tumors from 6 pigs (2 belonging to the R1 stage and 6 to the R2 stage). For each tumor, the four subsets are represented in the first two dimensions of the PCA. Confidence concentration ellipses are represented for each macrophage subset (confidence level set to 95%). **b, c** Relative gene expression for genes related to different functions showing differential expression within macrophages subsets (**b**) and for other genes statistically differently expressed in MHCII^+^ CD163^+^ macrophages (**c**). Relative gene expressions were calculated by subtracting the ΔCt(sample) from the ΔCt(mean) across all samples and the 2^−ΔΔCt^ method. Statistical analyses: One-way ANOVA followed by Tukey’s multiple comparison test are represented with bars (*p < 0.05, **p < 0.01). The statistical description of the macrophage subpopulations was also applied after a PCA (# mark when a subpopulation statistical differs from others subpopulations, see supplementary Table 9 for exact *v* test and *p* values)

To investigate their functionality, we sorted the four different subsets of TAMs from pigs belonging to R1 and R2 stages of regression and analyzed their gene expression profiles by RT-qPCR. Globally, MHCII^+^ CD163^−^ TAMs clustered and differed significantly from the other subsets (Fig. 6a). The detailed gene expression is presented in Fig. 6b for the genes for which a statistically significant difference could be revealed, others are in supplementary Fig. 2. MHCII^+^ CD163^−^ TAMs express lower levels of melanoma genes (*LYZ*, *S100A8*), cytokines and chemokines genes (*IL1B*, *IL8*, *IL10*, *CCL2* and *CCL5*), of antigen presentation related genes (*CD80* and *CD274*), and of genes implicated in angiogenesis, invasion and epithelial-mesenchymal transition (*CSF1*, *MMP14* and *VEGFA*). They show also lower expression of *ARG1* and *IDO1* (even though not significantly) and higher levels of *IL4I1*. MHCII^+^ CD163^+^ TAMs also differ from other TAMs subpopulations, with higher expression of *AIF*, *MPEG1*, *FCGR2B*, *VEGFA* and lower expression of *NOS2*, *MMP9* and *CSF1*. The increase of MHCII^+^ CD163^−^ TAMs, occurring concomitantly with the first signs of regression, and functionally very different from other TAMs subpopulations supports their contribution in the initiation of the regression process.

## Discussion

The activation of immune system plays a crucial role in the control of malignant cell growth in cutaneous melanoma. Nevertheless, the immune actors are in most cases inefficient with the exception of infrequent clinical situations characterized by partial or complete spontaneous tumor regression. The regression process is associated with an important tumor lymphoid infiltration [18]. Thus, CD8^+^ cytotoxic T cells are considered as the main effectors recognizing specifically tumor-associated antigens and their significant presence in melanoma lesions in regression supported their direct involvement in the immune-mediated regression [19, 20]. However, other analyses revealed no differences in the CD8^+^ T cell infiltration, when comparing either melanoma with or without regression, or regressed and not regressed areas from the same lesions [21, 22]. Such a result discrepancy may be due either to various parameters required for the regression classification or to the lesion analysis at different stages of the regression. Anyway, whether the CD8^+^ T cell infiltration is a cause or a consequence of tumor regression remains unclear. Beside CD8^+^ T cells, the contribution of CD56^+^ NK cells as cytotoxic effectors during early regression phase is supported by the activation of NK cell receptors in the tumor bed [23, 24] and the increased NK cell number in regressing melanocytic lesions [21]. Alternatively, NK cells might eliminate melanoma cells through the secretion of TNFα and IFNγ promoting Th1 differentiation and the recruitment of CD8^+^ T cells into regressing tumors [25, 26]. The number of global CD4^+^ T cells was similar in human melanoma lesions with or without any regression [22]. Among CD4^+^ T cells, FOXP3^+^ regulatory T cells are rare in areas with regression, and much more frequent in areas without regression [22, 27]. A recent study in the MeLiM pig model showed that CD4^−^CD8^+^ T cells accounted for the majority of tumor-infiltrating lymphocytes (TILs) during late phases of melanoma regression and that only CD4^+^CD8^hi^ TILs expanded during latest stages [28]. Here we deciphered more finely the kinetics of the initiation of regression. We found that neither CD4^+^CD8α^−^, CD4^+^CD8α^+^ and CD4^−^CD8α^+^ T cells, nor γδ T cells, were present in the active phase of the regression and accumulated only in late regression. This data supports the notion of a regression process initiated independently of these T cell subsets presence. We further reported that NK cells infiltrated tumors earlier than T cells consistent with their contribution in melanoma regression and their potential role in the recruitment of CD8^+^ T cells within the tumor microenvironment.

B cells are unique producers of antibodies, but several B-cell subsets can differently shape the responses to melanoma through their capacity to produce pro-inflammatory or anti-inflammatory cytokines and to present tumor-associated antigens [29]. Data on B cells infiltrating human melanoma regression are still limited, and their involvement in this process remains elusive. The number of global CD20^+^ B cells was similar in human melanoma lesions with or without any evidence of regression [22]. Even if B lymphocyte infiltration was very limited in MeLiM and restrained to the late phases of regression, we have previously reported an immunoglobulin signature [14] and that tumor regression is frequently accompanied by a local or systemic depigmentation of the skin, hair, and eyes that is associated with a *CD4* haplotype affecting the concentration of seric Ig [13].

Regarding the role of myeloid cells, human melanoma regression areas were characterized by a high number of DC that sustains their involvement in the spontaneous tumor cell destruction [27, 30]. In all studied MeLiM tumors, the proportion of different subtypes of DC remained low and quite stable arguing against crucial role for DC in melanoma regression in the pig model. In contrast, PMN cells decreased all along the regression process. Whether such a reduction contributes to the tumor control requires further investigations.

TAMs account for a substantial but variable proportion of the immune cell infiltrate in human cutaneous melanoma [31]. Even though TAMs promote tumor growth in many solid tumor types [32–34], they may also exhibit protective roles in specific disease stages or cancer types [35, 36]. Their significance for clinical outcome remains unclear probably due to the large spectrum of their phenotypes ranging from classically activated (or M1-like) and alternatively activated (or M2-like) associated with anti- and pro-tumoral functions, respectively, and their heterogeneity within the tumor microenvironment [37–39]. Thus, Etzerodt et al*.* recently identified in a melanoma mouse model four different TAM subsets depending on their expression of the scavenger receptor CD163 and MHCII (CD163^−^ MHCII^−^, CD163^−^ MHCII^+^, CD163^+^ MHCII^−^, CD163^+^ MHCII^+^) [40]. CD163^+^ TAMs represent the most mature and immunosuppressive, characterized by an increased expression of genes associated with a M2-like signature (including *Il4ra*, *Mrc1*, *Stab1* and *Slco2b1*) or T cell suppression (*Il10*, *Ido1* and *Lgals1*), and their specific depletion reduced significantly melanoma growth by driving the recruitment of inflammatory monocytes and subsequently CD4^+^ and CD8^+^ T cell recruitment and activation. Notably, CD163^+^ TAMs are associated with a poor prognosis in human melanoma [41].

Compared to the abundant knowledge of TAMs in tumorigenesis [34, 39], the role of the TAMs in tumor regression is only poorly investigated. For instance, TAMs can switch from pro-tumoral to anti-tumoral status and cooperate with TILs leading to reduced tumor burden after STING/Type 1 IFN activation or inhibition of Class IIa HDAC or Clever-1 [42–44]. Our data in the MeLiM model highlight for the first time the role of TAMs in the spontaneous melanoma regression. Indeed, they represented about 70% among tumor-infiltrating CD45^+^ cells from R0 to R3 stages. In particular, MHCII^+^ CD163^−^ TAMs were the earliest tumor-infiltrating immune population during the regression process that doubled between R0 and R1 lesions and significantly increased at R2 stage. More interestingly, this subset differed significantly from MHCII^+^ CD163^+^, MHCII^−^ CD14^−^ and MHCII^−^ CD14^+^ TAMs in R1 and R2 stages. It exhibited lower levels of *IL1B*, *IL8 *and *IL10* and *CCL5* genes and of genes implicated in angiogenesis (i.e. *VEGFa*). Their low expression of antigen presentation related genes (*CD80* and *CD274*) and the absence of T cells at the earliest stage of melanoma regression support a T-cell independent action of macrophages on tumor cells. Nonetheless, MHCII + CD163− TAMs accumulate within the regressing lesions (R3, R4) and could also promote the activation of tumor-specific T cells. Compared to other TAMs subsets, they display very low levels of *Arginase 1* and *IDO1* and a high level of *IL4I1* gene expression.

IL4I1 is a secreted l-amino acid oxidase that mainly catabolizes l-phenylalanine, known to be expressed by TAMs of most solid tumors [45, 46]. This enzyme promotes tumor growth by shaping the immune microenvironment in transplanted and spontaneous melanoma murine models [47, 48]. Our recent data also showed that IL4I1 expression is related to a microenvironment enriched in regulatory T cells and poor in granzyme B-positive CD8^+^ T cells in human primary cutaneous melanoma and is associated with a higher risk of poor outcome in patients [49]. Altogether, these data support a key role for IL4I1 in melanoma aggressiveness. The high transcriptional IL4I1 expression by MHCII^+^ CD163^−^ TAMs in MeLiM pigs with regressing melanoma was unexpected, but may result from the enriched IFNγ, TNFα, IL12 and IL6 tumor microenvironment between R0 and R1 stages. Interestingly, programmed cell death 1 receptor is a marker of both activation and exhaustion of melanoma-infiltrating T cells. Whether IL4I1 expression impacts the properties of TAMs similarly requires further investigations. The conflicting effects of the IL4I1 enzyme may depend on the tumor context, either deleterious in the context of a chronic inflammation (tumor progression) or anti-tumoral in the context of tumor regression. Our present data shows that IL4I1 produced by MHCII^+^ CD163^−^ TAMs does not prevent tumor control in our model. This enzyme, known for its primarily immunosuppressive effect on T cells, may even contribute to the regression of melanoma, at the earliest stages of the process when T cells are absent from the melanoma microenvironment. Finally, it is crucial to unravel whether IL4I1 derived from MHCII^+^ CD163^−^ TAMs initiates the tumor control or is merely a consequence of the tumor regression to develop an accurate therapy targeting this enzyme.

In summary, spontaneously regressing melanoma represents a fascinating model to improve our knowledge in the natural immune process of tumor clearance. Our study clearly identified distinct waves of immune cells from the earliest stage of regression to the latest one and evidenced the presence of a particular TAMs subset in very early regressing lesions. Although current immunotherapies mainly aim to reinvigorating the TILs activity, harnessing both the adaptive and innate arms of the immune system emerged to improve treatment. In particular, the appropriate activation of MHCII^+^ CD163^−^ TAMs might lead to more efficient treatment for melanoma.

### Supplementary Information

Below is the link to the electronic supplementary material.

Supplementary file1 (PDF 1349 kb)

## Data Availability

The data generated in this study are available within the article and its supplementary data files.

## Abbreviations

FAMD: Factorial analysis of mixed data
MeLiM: Melanoma-bearing Libechov Minipigs
PCA: Principal component analysis
TAM: Tumor-associated macrophage
TIL: Tumor-infiltrating lymphocyte
