# Supplementary material for: Deciphering the immune reaction leading to spontaneous melanoma regression: initial role of MHCII+ CD163− macrophages
Source: Cancer Immunol Immunother. 2023 Aug 1;72(11):3507–21. doi: 10.1007/s00262-023-03503-6 (PMC10576715; doi:10.1007/s00262-023-03503-6)
Supplement: Supplementary file 1 — Supplementary file1 (PDF 1349 kb) [file 262_2023_3503_MOESM1_ESM.pdf]

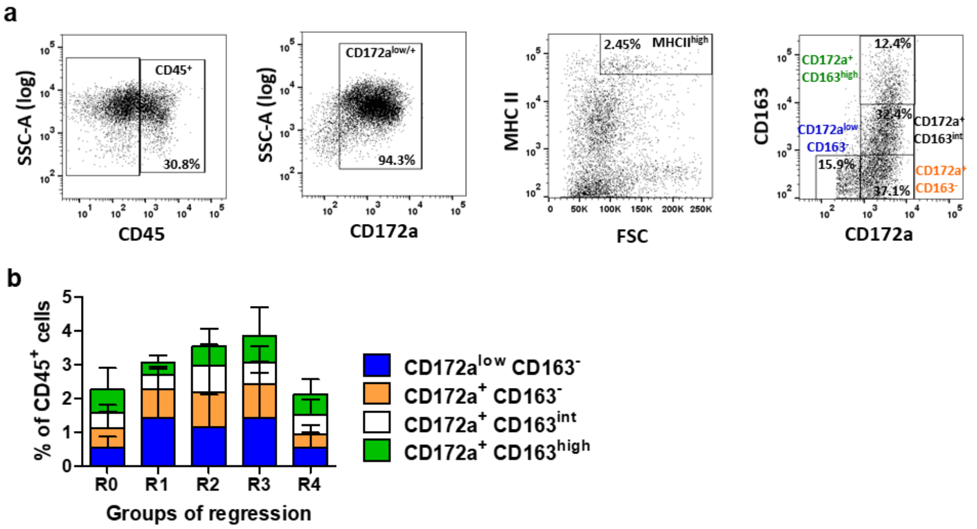

Supplementary Fig. 1 Dendritic cells in melanoma lesions along the regression process. a Gating strategy to identify tumor infiltrating DCs. Cells were first gated on the CD45^{+} population. CD172a$^{low/+}$ cells were then selected and DCs were identified as FSC^{high} MHCII^{high} cells within the CD45^{+} CD172a$^{low/+}$ cells. Four subsets were then determined based on their expression of CD172a and CD163. Percentages of the parent populations are shown on each dot plot representing 10000 events. **b** Proportion of the different DC subsets among tumor-infiltrating CD45^{+} cells. In the plots, bars represent the means +/- SEM. (n=16 for R0, n=15 for R1, n=20 for R2, n=17 for R3 and n=14 for R4).

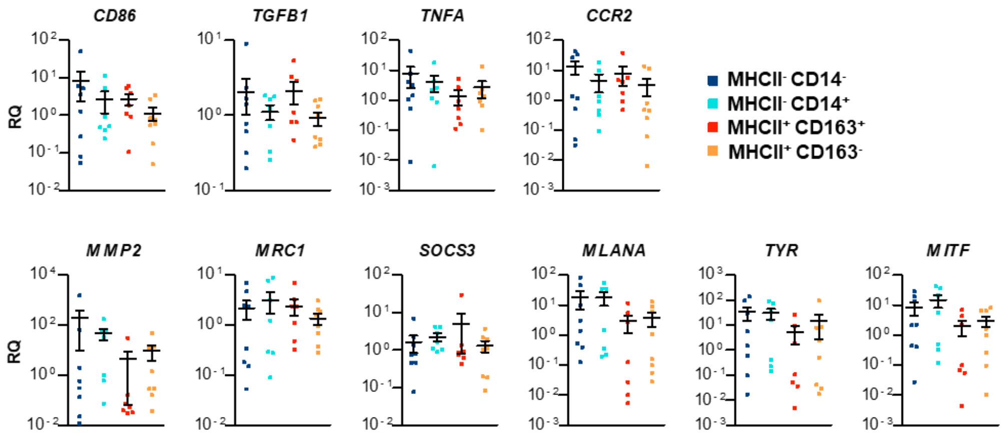

Supplementary Fig. 2 Gene expression analysis from 4 TAMs subsets: MHCII^{-} CD14^{-}, MHCII^{-} CD14^{+}, MHCII^{+} CD163^{-}, and MHCII^{+} CD163^{+}. Relative gene expression were calculated by subtracting the ΔCt(sample) from the ΔCt(mean) across all samples and the $2^{-\Delta\Delta Ct}$ method.

Supplementay table 1: Pig's individual clinical data and metadata
Tumor burden is defined as low if total number of lesions is comprised between 1 and 3, medium if between 4 and 7 and high if above 8.

Pig	Litter	Sex	*CD4* genotype	Number of lesions		tumor burden	Death (day)	Palpable lymphadenopathies		Lesions excised	
				At birth	Total			Observation	Day of appearance	Number	Age of the pig (day)
1331	159	F	Ref/Alt	9	14	high	401	observed	30	8	16, 36, 58, 79, 100, 121, 142, 154
1335	159	F	Ref/Ref	5	8	high	1655	observed	22	5	36, 58, 79, 100, 121
1336	159	F	Ref/Alt	4	7	medium	346	observed	22	6	16, 36, 58, 79, 121, 154
1341	160	M	Ref/Ref	2	3	low	471	observed	14	2	34, 56
1343	160	F	Ref/Alt	4	6	medium	1064	observed	84	2	14, 56
1362	162	F	Ref/Alt	1	12	high	1014	observed	32	4	40, 61, 82, 94
1364	163	M	Ref/Alt	6	11	high	739	observed	26	5	39, 60, 72, 107, 130
1367	163	F	Ref/Alt	2	7	medium	992	observed	38	4	39, 60, 72, 107
1376	164	M	Ref/Ref	1	9	high	301	observed	42	1	42
1377	164	F	Ref/Alt	6	9	high	645	observed	36	4	21, 41, 54, 89
1378	164	F	Ref/Ref	1	10	high	378	observed	42	2	21, 54
1387	165	M	Ref/Ref	4	4	low	415	non-observed		3	29, 49, 69
1396	166	M	Ref/Alt	3	4	low	301	observed	20	2	28, 68
1397	166	F	Ref/Alt	2	9	high	1471	observed	20	1	48
1399	166	M	Ref/Ref	2	4	low	68	observed	27	1	28
1467	173	F	Ref/Alt	4	6	medium	319	observed	36	1	79
1470	174	M	Ref/Ref	2	5	medium	634	observed	33	3	19, 54, 89
1472	174	F	Ref/Ref	4	7	medium	832	observed	33	3	19, 54, 89
1473	174	F	Ref/Ref	2	5	medium	622	observed	56	3	19, 54, 89
1474	174	F	Ref/Ref	5	9	high	712	observed	26	3	19, 54, 89
1475	174	F	Ref/Ref	5	9	high	832	observed	33	3	19, 54, 89
1479	175	M	Ref/Alt	3	9	high	1757	observed	13	3	54, 82, 105
1480	175	M	Ref/Alt	1	4	low	322	observed	48	1	105
1482	175	M	Ref/Alt	1	8	high	119	observed	27	1	82
1483	175	F	Ref/Alt	2	3	low	322	observed	13	2	54, 105
1489	176	F	Ref/Ref	2	3	low	290	observed	45	1	51
1490	176	F	Ref/Alt	1	11	high	890	observed	31	2	51, 73
1506	178	M	Ref/Ref	4	9	high	1278	observed	105	3	28, 69, 69
1510	178	F	Ref/Ref	2	7	medium	1029	observed	105	2	69, 69
1512	178	F	Ref/Ref	6	10	high	797	observed	105	1	28
1516	179	F	Alt/Alt	8	14	high	379	observed	44	6	48, 48, 48, 48, 48, 55
1517	179	M	Alt/Alt	1	3	low	134	observed	44	1	55
1518	179	M	Ref/Alt	2	4	low	134	observed	44	1	48
1519	179	M	Ref/Alt	1	6	medium	1400	observed	44	2	48, 55

Supplementay table 2: Clinical and histological phenotypes for each tumoral

ND: not determined

							Clinical obervations of the lesions						Histological obervations of the tumoral zone											Histological obervations of the regressive zone		
Pig_Lesion	Pig	Lesion	Sex	Age of the pig at excision (day)	Presence of a zone of dermal fibrosis	Stage of regression	Lesion size (cm) (Clin_Size)	Lesion size's evolution (Clin_Size_Evolution)	Description (Clin _Description)	Ulceration (Clin _Ulceration)	Grayish lesion (Clin _Grayish)	Palpable adenomegalies (Clin _Adenomegalies)	Profile (Histo_Profile)	Length (cm)	Depht (cm)	Area (mm^2)	Clark's level (Histo_Clark)	Presence of Grenz Zone (Histo_Grenz_Zone)	Percentage of connective tissue (Histo_Connective_Tissue)	Ulceration (Histo_Ulceration)	Vascularization (Histo_T _Vascularization)	Clusters of melanophages (Histo_Clustered_melanophages)	Lymphoid infiltration (Histo_T_Lymphoid _Infiltration)	Extension (Histo_R_Extension)	Neoangiogenesis (Histo_R _Neoangiogenesis)	Lymphoid infiltration (Histo_R_Lymphoid_Infiltration)
1331_L3	1331	L3	F	16	no	R0	2.5	increasing	dome	yes (++)	no	no	dome or plateau	0.74	1.08	50.9	V	no	6.6	yes	high	no	yes (perilesional)	-	-	-
1336_L2	1336	L2	F	16	no	R0	2.7	increasing	plateau	no	no	yes	dome or plateau	1.66	0.49	55.9	V	no	7.1	yes	high	no	yes (perilesional)	-	-	-
1343_L1	1343	L1	F	14	no	R0	3.0	increasing	dome	yes (++)	no	no	polypoid	1.93	0.71	124.4	V	no	3.9	yes	high	no	no	-	-	-
1376_L1	1376	L1	M	42	no	R0	2.5	increasing	plateau	yes (+)	no	yes	dome or plateau	2.45	0.97	191.1	V	no	8.5	yes	high	no	no	-	-	-
1377_L3	1377	L3	F	21	no	R0	1.5	increasing	dome	no	no	no	ND	0.56	0.28	10.7	ND	no	10.3	no	high	no	no	-	-	-
1378_L1	1378	L1	F	21	no	R0	2.3	increasing	plateau	no	no	no	dome or plateau	1.86	0.94	128.3	V	no	2.8	yes	high	no	no	-	-	-
1387_L2	1387	L2	M	29	no	R0	2.0	increasing	plateau	no	no	no	dome or plateau	1.66	0.52	66.4	V	no	7.3	yes	high	no	no	-	-	-
1396_L2	1396	L2	M	28	no	R0	2.5	stable	plateau	yes (+)	no	yes	dome or plateau	2.10	0.61	92.0	V	no	10.2	yes	high	no	no	-	-	-
1399_L1	1399	L1	M	28	no	R0	3.3	increasing	plateau	yes (+)	no	yes	dome or plateau	2.36	0.69	130.8	V	no	5.2	yes	high	no	no	-	-	-
1472_L2	1472	L2	F	19	no	R0	3.9	increasing	dome	yes (++)	no	no	polypoid	3.77	1.25	344.0	V	no	4.0	yes	high	no	no	-	-	-
1474_L2	1474	L2	F	19	no	R0	2.5	increasing	dome	yes (++)	no	no	polypoid	2.82	1.17	184.9	V	no	5.9	yes	high	no	no	-	-	-
1475_L2	1475	L2	F	19	no	R0	3.0	increasing	dome	yes (+)	no	no	polypoid	2.23	0.71	155.3	IV	no	11.6	yes	high	no	no	-	-	-
1519_L3	1519	L3	M	48	no	R0	4.7	increasing	dome	yes (++)	no	yes	dome or plateau	4.12	1.55	496.3	V	no	2.8	yes	high	no	yes (intralesional)	-	-	-
1331_L8	1331	L8	F	36	yes	R1	2.9	stable	dome	yes (+)	no	yes	dome or plateau	1.90	1.01	119.3	IV	no	8.6	yes	high	yes	yes (perilesional)	dermal	yes	no
1335_L2	1335	L2	F	36	yes	R1	2.5	stable	dome	yes (+)	no	yes	polypoid	ND	ND	ND	V	no	4.0	yes	high	yes	no	dermal	no	no
1336_L4	1336	L4	F	36	yes	R1	2.8	increasing	dome	yes (+)	no	yes	polypoid	1.09	1.10	101.5	V	no	21.7	yes	high	yes	yes (intralesional)	dermal	yes	yes
1341_L3	1341	L3	M	34	yes	R1	2.5	increasing	dome	no	no	yes	dome or plateau	0.78	1.02	77.3	ND	no	2.1	yes	high	yes	no	dermal	no	no
1362_L4	1362	L4	F	40	yes	R1	2.3	increasing	plateau	no	no	yes	dome or plateau	1.55	0.52	65.5	V	no	17.5	yes	high	yes	no	dermal	yes	no
1364_L1	1364	L1	M	39	yes	R1	5.1	increasing	dome	yes (+)	no	yes	polypoid	3.33	1.65	303.3	V	no	20.0	yes	high	yes	yes (intralesional)	dermal	yes	no
1367_L2	1367	L2	F	39	yes	R1	2.7	increasing	dome	yes (+)	no	yes	dome or plateau	2.36	1.16	211.6	V	no	22.9	yes	high	yes	no	dermal	yes	no
1377_L6	1377	L6	F	42	yes	R1	2.3	stable	plateau	yes (++)	no	yes	polypoid	1.52	0.68	74.5	IV	no	24.1	yes	high	yes	yes (perilesional)	dermal	yes	no
1387_L1	1387	L1	M	49	yes	R1	2.0	stable	plateau	no	yes	no	dome or plateau	0.79	0.48	25.7	V	no	29.5	no	high	yes	no	dermal	no	no
1397_L2	1397	L2	F	48	yes	R1	3.2	stable	dome	yes (+)	no	yes	dome or plateau	1.26	0.91	89.2	V	no	21.1	yes	high	yes	no	dermal	yes	no
1470_L1	1470	L1	M	19	yes	R1	2.8	increasing	dome	yes (++)	no	no	dome or plateau	2.13	0.46	78.2	IV	no	7.0	yes	high	yes	no	dermal	no	no
1473_L1	1473	L1	F	19	yes	R1	3.0	increasing	exophytic	yes (+)	no	no	polypoid	2.48	1.43	217.3	V	no	28.0	yes	high	yes	no	dermal	no	no
1506_L3	1506	L3	M	28	yes	R1	3.5	increasing	exophytic	yes (+)	no	yes	polypoid	2.26	0.96	176.8	V	no	12.0	yes	high	yes	no	dermal	yes	no
1512_L1	1512	L1	F	28	yes	R1	2.9	increasing	exophytic	yes (+)	no	yes	polypoid	2.04	0.77	132.0	V	no	15.5	yes	high	yes	yes (perilesional)	dermal	yes	no
1516_L1	1516	L1	F	48	yes	R1	3.6	stable	dome	no	no	yes	polypoid	2.67	1.17	234.2	V	no	10.4	yes	high	yes	no	dermal	yes	no
1516_L11	1516	L11	F	48	yes	R1	2.1	increasing	plateau	yes (+)	no	yes	dome or plateau	1.39	0.74	74.6	V	no	12.1	yes	high	yes	no	ND	no	no
1516_L2	1516	L2	F	48	yes	R1	3.3	increasing	dome	no	no	yes	polypoid	2.20	1.05	178.9	V	no	22.4	yes	high	yes	no	dermal	yes	yes
1516_L7	1516	L7	F	48	yes	R1	2.9	increasing	dome	yes (++)	no	yes	polypoid	1.70	1.04	139.6	V	no	13.1	yes	high	yes	yes (perilesional)	dermal	yes	no
1516_L9	1516	L9	F	48	yes	R1	3.4	stable	plateau	no	no	yes	polypoid	2.59	0.84	150.7	V	no	15.7	yes	high	yes	no	dermal	yes	yes
1518_L1	1518	L1	M	48	yes	R1	4.9	stable	dome	no	no	yes	polypoid	3.60	1.56	384.8	V	no	26.4	yes	high	yes	yes (intralesional)	dermal	yes	yes
1331_L9	1331	L9	F	58	yes	R2	2.3	increasing	exophytic	yes (+)	no	yes	dome or plateau	1.59	0.74	86.5	IV	no	14.2	yes	high	yes	yes (perilesional)	dermal	no	no
1335_L1	1335	L1	F	58	yes	R2	2.7	increasing	dome	yes (++)	no	yes	dome or plateau	1.37	0.30	38.3	ND	no	12.4	yes	high	yes	no	dermal	yes	no
1336_L5	1336	L5	F	58	yes	R2	3.2	increasing	plateau	no	yes	yes	dome or plateau	2.08	0.57	106.8	IV	no	23.2	yes	high	yes	yes (intralesional)	dermal	yes	yes
1341_L1	1341	L1	M	56	yes	R2	2.5	increasing	plateau	no	yes	yes	polypoid	1.77	1.31	169.9	V	no	14.5	no	high	yes	no	dermal	yes	no
1343_L4	1343	L4	F	56	yes	R2	1.4	stable	dome	no	no	no	polypoid	0.97	0.34	31.7	IV	no	9.6	yes	high	yes	no	dermal	yes	no
1362_L1	1362	L1	F	61	yes	R2	3.3	increasing	plateau	no	no	yes	dome or plateau	1.98	0.89	146.0	V	no	14.9	yes	low	yes	no	dermal	yes	yes
1364_L3	1364	L3	M	60	yes	R2	3.6	stable	dome	no	yes	yes	polypoid	2.19	1.72	241.5	V	no	25.2	yes	high	yes	yes (intralesional)	dermal and epidermal	yes	yes
1367_L4	1367	L4	F	60	yes	R2	2.0	stable	dome	no	no	yes	polypoid	1.82	1.22	166.3	V	no	21.5	yes	high	yes	no	dermal and epidermal	yes	no
1377_L5	1377	L5	F	54	yes	R2	2.2	stable	plateau	no	yes	yes	dome or plateau	2.05	0.93	149.0	IV	no	23.1	no	high	yes	yes (perilesional)	dermal	yes	yes
1378_L3	1378	L3	F	54	yes	R2	3.0	increasing	plateau	yes (+)	no	yes	dome or plateau	3.81	0.98	255.8	V	no	18.0	yes	high	yes	no	dermal	yes	no
1387_L3	1387	L3	M	69	yes	R2	2.0	stable	plateau	no	yes	no	dome or plateau	1.49	0.94	97.1	IV	no	9.5	no	high	yes	yes (intralesional)	dermal	yes	no
1396_L4	1396	L4	M	68	yes	R2	2.0	stable	plateau	no	no	yes	dome or plateau	1.21	0.27	22.5	V	no	19.3	no	high	yes	no	dermal	yes	no
1470_L3	1470	L3	M	54	yes	R2	2.1	stable	plateau	no	no	yes	dome or plateau	0.95	0.45	28.7	IV	no	21.2	no	high	yes	no	dermal	yes	no
1472_L1	1472	L1	F	54	yes	R2	3.9	stable	dome	yes (+)	no	yes	dome or plateau	1.98	1.64	253.9	V	yes	9.2	yes	high	yes	yes (intralesional)	dermal	yes	no
1473_L2	1473	L2	F	54	yes	R2	2.7	stable	plateau	no	yes	no	dome or plateau	1.27	0.78	86.3	V	no	25.4	no	high	yes	yes (intralesional)	dermal and epidermal	yes	yes
1474_L3	1474	L3	F	54	yes	R2	2.2	stable	plateau	no	yes	yes	dome or plateau	1.51	0.52	56.0	IV	no	55.0	no	high	yes	yes (intralesional)	dermal and epidermal	yes	yes
1475_L3	1475	L3	F	54	yes	R2	2.9	increasing	plateau	yes (++)	no	yes	dome or plateau	1.97	0.50	50.3	V	no	29.5	yes	high	yes	yes (intralesional)	dermal	yes	no
1479_L1	1479	L1	M	54	yes	R2	4.8	stable	dome	yes (+)	no	yes	polypoid	3.21	1.49	342.0	IV	no	35.8	yes	high	yes	yes (perilesional)	dermal	yes	yes
1483_L2	1483	L2	F	54	yes	R2	2.7	stable	plateau	no	no	yes	polypoid	1.66	0.70	83.9	IV	no	39.7	yes	high	yes	yes (perilesional)	dermal	yes	no
1489_L3	1489	L3	F	51	yes	R2	3.1	stable	plateau	no	no	yes	dome or plateau	2.13	1.29	159.1	V	no	10.2	yes	high	yes	no	dermal	yes	yes
1490_L1	1490	L1	F	51	yes	R2	3.1	increasing	plateau	yes (+)	no	yes	polypoid	2.59	0.95	160.8	V	no	11.3	yes	high	yes	no	ND	yes	no
1506_L1	1506	L1	M	69	yes	R2	3.2	increasing	plateau	no	no	yes	dome or plateau	2.33	0.58	93.8	IV	no	12.0	yes	high	yes	no	dermal	no	no
1506_L2	1506	L2	M	69	yes	R2	3.3	stable	dome	yes (+)	no	yes	dome or plateau	2.35	1.31	223.4	V	no	14.8	yes	high	yes	no	dermal	yes	no
1510_L2	1510	L2	F	69	yes	R2	3.0	stable	plateau	no	no	yes	dome or plateau	1.62	0.90	98.8	V	no	22.3	no	high	yes	yes (perilesional)	dermal	yes	yes
1510_L7	1510	L7	F	69	yes	R2	2.8	stable	plateau	yes (+)	no	yes	dome or plateau	1.98	0.91	118.6	IV	no	8.9	yes	high	yes	yes (perilesional)	dermal	yes	no
1516_L8	1516	L8	F	55	yes	R2	2.8	increasing	plateau	no	no	yes	dome or plateau	1.56	0.86	96.7	V	no	9.2	yes	high	yes	no	dermal	no	no
1517_L1	1517	L1	M	55	yes	R2	4.7	increasing	exophytic	yes (+)	no	yes	polypoid	2.80	1.63	290.8	IV	no	11.6	yes	high	yes	yes (perilesional)	dermal	yes	no
1519_L2	1519	L2	M	55	yes	R2	3.6	stable	plateau	yes (+)	no	yes	dome or plateau	3.20	1.30	300.7	V	no	9.8	yes	low	yes	yes (perilesional)	dermal	no	no
1331_L4	1331	L4	F	79	yes	R3	2.6	stable	dome	no	yes	yes	dome or plateau	2.33	0.92	172.8	IV	no	18.9	yes	high	yes	yes (intralesional)	ND	yes	yes
1335_L5	1335	L5	F	79	yes	R3	2.4	stable	dome	no	yes	yes	dome or plateau	ND	ND	ND	IV	no	20.9	yes	high	yes	no	dermal	yes	yes
1336_L3	1336	L3	F	79	yes	R3	2.3	decreasing	plateau	no	yes	yes	dome or plateau	1.96	1.65	247.0	V	no	25.5	yes	high	yes	yes (intralesional)	dermal	yes	yes
1362_L3	1362	L3	F	82	yes	R3	2.7	stable	plateau	no	no	yes	dome or plateau	ND	ND	ND	V	no	28.6	no	high	yes	no	dermal	yes	yes
1362_L5	1362	L5	F	94	yes	R3	1.7	stable	plateau	no	yes	yes	dome or plateau	1.42	0.64	69.6	V	no	25.9	no	high	yes	yes (intralesional)	dermal	yes	yes
1364_L4	1364	L4	M	72	yes	R3	3.3	stable	dome	no	yes	yes	dome or plateau	4.06	0.94	273.8	V	no	24.9	no	high	yes	yes (intralesional)	dermal and epidermal	yes	yes
1367_L6	1367	L6	F	72	yes	R3	1.8	stable	plateau	no	yes	yes	dome or plateau	1.05	0.48	39.5	IV	no	17.3	no	high	yes	yes (intralesional)	dermal	no	yes
1377_L4	1377	L4	F	89	yes	R3	2.0	stable	exophytic	no	no	yes	polypoid	1.42	0.81	70.1	IV	no	21.3	yes	high	yes	yes (perilesional)	dermal	yes	yes
1467_L3	1467	L3	F	79	yes	R3	4.2	increasing	plateau	yes (+)	yes	yes	dome or plateau	3.60	1.58	432.4	V	no	34.6	yes	high	yes	no	dermal	yes	yes
1470_L2	1470	L2	M	89	yes	R3	2.5	stable	plateau	no	no	yes	dome or plateau	1.05	0.68	57.8	IV	no	29.9	no	high	yes	yes (perilesional)	dermal and epidermal	yes	yes
1472_L5	1472	L5	F	89	yes	R3	1.7	decreasing	plateau	no	yes	yes	dome or plateau	1.55	0.77	85.6	V	no	23.6	no	high	yes	no	dermal and epidermal	yes	no
1473_L3	1473	L3	F	89	yes	R3	2.1	decreasing	plateau	no	yes	yes	dome or plateau	1.13	0.48	41.2	V	yes	51.0	no	high	yes	yes (intralesional)	dermal and epidermal	yes	yes
1474_L8	1474	L8	F	89	yes	R3	2.6	stable	plateau	no	yes	yes	dome or plateau	0.69	0.40	23.4	V	ND	54.4	no	ND	yes	ND	dermal and epidermal	yes	yes
1475_L8	1475	L8	F	89	yes	R3	1.7	stable	dome	no	yes	yes	dome or plateau	1.43	0.84	101.1	IV	no	21.9	no	high	yes	yes (intralesional)	dermal and epidermal	yes	yes
1479_L3	1479	L3	M	82	yes	R3	4.2	stable	dome	no	yes	yes	dome or plateau	2.45	1.71	311.2	IV	no	30.9	no	high	yes	yes (intralesional)	dermal and epidermal	yes	yes
1482_L4	1482	L4	M	82	yes	R3	2.5	stable	plateau	no	no	yes	dome or plateau	1.90	0.50	51.0	V	no	15.0	no	high	yes	yes (intralesional)	dermal and epidermal	yes	yes
1490_L2	1490	L2	F	73	yes	R3	3.1	stable	dome	no	yes	yes	polypoid	1.56	1.54	172.5	IV	no	24.7	yes	high	yes	yes (intralesional)	dermal	yes	yes
1331_L2	1331	L2	F	121	yes	R4	3.2	stable	plateau	no	yes	yes	dome or plateau	2.26	1.17	206.0	V	yes	28.5	no	high	yes	yes (intralesional)	dermal and epidermal	yes	yes
1331_L5	1331	L5	F	142	yes	R4	2.8	decreasing	plateau	no	yes	yes	dome or plateau	ND	ND	ND	IV	yes	31.9	no	high	yes	yes (intralesional)	dermal and epidermal	yes	yes
1331_L6	1331	L6	F	100	yes	R4	1.7	stable	plateau	no	yes	yes	dome or plateau	1.35	0.74	75.5	IV	no	29.5	no	high	yes	yes (perilesional)	dermal and epidermal	yes	no
1331_L7	1331	L7	F	154	yes	R4	2.5	decreasing	plateau	no	yes	yes	dome or plateau	1.93	0.43	72.9	IV	yes	39.1	no	high	yes	yes (intralesional)	dermal and epidermal	yes	yes
1335_L3	1335	L3	F	100	yes	R4	1.8	increasing	plateau	no	yes	yes	dome or plateau	1.38	0.57	66.0	IV	no	30.1	yes	high	yes	yes (intralesional)	dermal	yes	no
1335_L6	1335	L6	F	121	yes	R4	1.8	stable	plateau	no	yes	yes	dome or plateau	1.94	0.74	89.6	IV	no	35.0	yes	high	yes	yes (perilesional)	dermal	yes	yes
1336_L1	1336	L1	F	121	yes	R4	3.2	increasing	plateau	no	yes	yes	dome or plateau	0.95	0.64	57.2	V	yes	37.0	no	high	yes	yes (perilesional)	dermal and epidermal	yes	yes
1336_L7	1336	L7	F	154	yes	R4	1.4	stable	plateau	no	yes	yes	dome or plateau	1.04	0.42	33.2	IV	yes	51.0	no	high	yes	yes (perilesional)	dermal and epidermal	no	yes
1364_L2	1364	L2	M	107	yes	R4	2.7	decreasing	dome	no	yes	yes	dome or plateau	1.80	1.91	259.1	V	yes	34.7	no	high	yes	yes (intralesional)	dermal and epidermal	yes	yes
1364_L6	1364	L6	M	130	yes	R4	1.5	stable	dome	no	yes	yes	dome or plateau	1.34	0.85	95.0	IV	yes	25.5	no	high	yes	yes (intralesional)	dermal and epidermal	yes	yes
1367_L5	1367	L5	F	107	yes	R4	2.2	stable	plateau	no	yes	yes	dome or plateau	2.61	1.16	244.1	V	yes	37.5	yes	high	yes	yes (perilesional)	dermal and epidermal	yes	yes
1479_L4	1479	L4	M	105	yes	R4	2.2	stable	plateau	no	yes	yes	dome or plateau	1.36	0.17	20.9	V	yes	53.2	no	ND	yes	ND	dermal and epidermal	yes	yes
1480_L1	1480	L1	M	105	yes	R4	2.2	stable	plateau	no	yes	yes	dome or plateau	1.25	1.20	109.5	V	yes	20.3	no	high	yes	yes (perilesional)	dermal and epidermal	yes	yes
1483_L1	1483	L1	F	105	yes	R4	3.4	stable	dome	no	no	yes	dome or plateau	2.86	2.02	372.9	V	no	28.7	yes	high	yes	yes (perilesional)	dermal	yes	yes

Supplementay table 3: Cytokines assessed in tumoral lesions (expressed in pg/mg of tumor).

ND: not determined

Pig_Lesion	Stage of regression	IL-1β	IL-2	IL-4	IL-6	IL-8	IL-10	IL-12	IFNγ	TNFα	CCL2	TGFβ	IL-17	IFNα
1331_L3	R0	11426	28972	980	8935	1030	6443	6157	9202	17150	2216	742	108.6	606.0
1331_L4	R3	2141	4407	132	547	263	2409	1102	3953	4541	ND	ND	0.0	32.5
1331_L6	R4	7123	15450	632	3242	662	4046	3496	6613	11240	2256	644	63.9	316.8
1331_L9	R2	20077	6998	219	963	1962	2960	957	4595	5242	4178	692	26.6	49.3
1335_L1	R2	15008	4464	169	702	1504	1420	712	4144	2531	5940	1126	111.1	28.3
1335_L2	R1	30427	4823	236	586	1590	773	904	4146	2910	4780	870	134.0	75.4
1335_L3	R4	3891	8245	334	1401	379	1880	1953	5283	5098	1938	472	9.6	125.4
1335_L5	R3	4867	11452	408	2221	412	2751	2555	6058	4323	2446	746	31.5	183.0
1336_L1	R4	1287	1569	56	121	124	606	527	2900	664	334.4	212	0.0	0.0
1336_L2	R0	19079	6147	169	749	3254	2636	719	4298	4120	3370	644	52.8	30.9
1336_L3	R3	7584	12841	504	2622	503	3903	3123	6398	7807	1670	606	39.4	217.5
1336_L5	R2	6407	8187	307	1305	506	2320	1262	5060	5059	3588	792	32.6	88.0
1343_L1	R0	6279	15452	551	2943	615	3170	3472	6687	8610	ND	ND	54.5	271.1
1343_L4	R2	7324	15205	501	3311	673	3476	3398	6887	10015	2654	774	58.2	242.7
1362_L3	R3	10667	22257	835	5112	837	4957	4668	7560	12566	1740	612	63.4	444.5
1362_L4	R1	10450	23934	814	7007	907	5340	4821	8921	15285	2806	812	70.1	458.0
1362_L5	R3	11454	16155	659	3311	676	3856	3072	6977	11152	1778	520	52.4	244.2
1364_L1	R1	10314	23289	813	6377	828	5078	6318	8721	17105	2302	1276	82.3	416.8
1364_L4	R3	14694	31681	1024	8560	935	6805	6395	9577	19226	2050	576	85.5	567.3
1367_L2	R1	8996	20331	707	5677	752	5451	4823	8117	14340	2828	670	62.4	391.5
1367_L4	R2	5598	11079	418	2105	497	3156	2032	5935	7607	2854	528	34.7	153.4
1367_L5	R4	2443	3603	136	383	188	597	726	3587	1588	450	574	0.0	21.5
1367_L6	R3	6445	8487	320	1639	635	2963	2764	5401	9063	4480	576	28.5	131.5
1377_L3	R0	7221	20522	602	7291	954	5570	5538	9286	12508	2446	718	46.1	363.4
1377_L4	R3	14381	9755	302	1813	1123	3429	1771	5618	6973	6760	640	120.8	112.4
1377_L5	R2	9862	20589	656	3864	643	5052	4836	7455	10500	2942	640	53.1	367.7
1378_L3	R2	8280	16337	569	4702	679	4589	3719	7758	10753	4110	542	59.7	294.3
1387_L1	R1	8265	19338	688	5854	728	4142	4258	8324	11374	2536	882	53.3	380.7
1387_L2	R0	10728	21275	664	5260	1449	4525	4397	7842	12318	1960	912	75.4	387.0
1397_L2	R1	4859	4397	141	556	846	1652	726	3703	3248	4660	1038	20.3	32.1
1470_L2	R3	1043	1938	87	105	89	576	472	2918	1024	ND	ND	0.0	0.0
1472_L1	R2	4217	2630	81	427	226	1930	706	3702	2521	670	702	0.0	17.6
1472_L2	R0	8585	18651	682	4956	839	4271	4336	7752	11670	ND	ND	90.0	357.5
1472_L5	R3	4591	12195	397	2974	659	3519	4332	6548	9966	2800	640	27.1	176.1
1474_L2	R0	8253	18338	664	5343	990	4301	4291	8078	11531	2052	702	86.2	86.2
1475_L2	R0	7057	14724	534	2590	581	3280	3563	6544	8060	ND	ND	48.1	48.1

Supplementary table 4: Antibodies used in this study.

A, B and C correspond to the three antibody combinations to identify cell subsets: A for lymphocyte subsets, B for B lymphocytes and C for myeloid cells. Combination D was used to analyze NK cell functionality. (* intracellular stainings)

Specificities	Clones / references	Isotypes	Target species	Fluorochrome	Labelling strategy	Providers	Working dilutions or concentrations	Used in combinations
CD45	K252- 1E4	m IgG1	pig	AF647	Directly conjugated	AbD Serotec	1/20	A, B, C, D
CD3	PPT3	m IgG1	pig	FITC	Directly conjugated	SouthernBiotech	2.5 µg/mL	A
CD8α	76-2-11	m IgG2a	pig	PE-Cy5	Directly conjugated	Abcam	1 µg/mL	A, D
CD4	74-12-4	m IgG2b	pig	APC-Cy7	Secondary antibody[a]	Abcam	2.5 µg/mL	A
gdTCR	PGBL22A	m IgG1	pig	PE-Cy7	Secondary antibody[b]	WSU	2 µg/mL	A
CD16	G7	m IgG1	pig	PE	Directly conjugated	AbD Serotec	1/20	A
MHCII	MSA3	m IgG2a	pig	AF488	Secondary antibody[c]	WSU	2 µg/mL	B
CD21	B-Ly4	m IgG1	human	PE-Cy7	Secondary antibody[b]	BD Biosciences	4 µg/mL	B
CD79a*	HM57	m IgG1	human	PE	Directly conjugated	AbD Serotec	50 µg/mL	B
control	MOPC-21	m IgG1		PE	Directly conjugated	Abcam	1/20	B
PG68A	PG68A	m IgG1	pig	PE-Cy7	Secondary antibody[b]	WSU	5 µg/mL	C
CD163	2A10/11	m IgG1	pig	PE	Directly conjugated	AbD Serotec	1/10	C
CD172a	74-22-15A	m IgG2b	pig	APC-Cy7	Secondary antibody[a]	WSU	1 µg/mL	C
CD14	TUK4	m IgG2a	human	Pacific Blue	Directly conjugated	AbD Serotec	1/10	C
CADM1	3E1	c IgY	human/ mouse	Qdot655	Secondary antibody[d]	MBL	4 µg/mL	C
CD3	8E6-8C8	m IgG2a	pig	PerCp-eFluor710	Secondary antibody[e]	WSU	5 µg/mL	D
CD16	G7	m IgG1	pig	PE-Cy7	Secondary antibody[b]	AbD Serotec	2.5 µg/mL	D
IFNγ*	P2G10	m IgG1	pig	PE	Directly conjugated	BD Biosciences	0.4 µg/mL	D
control	MOPC-21	m IgG1		PE	Directly conjugated	Abcam	1/500	D

[a] Goat anti-mouse IgG2b-APC-Cy7, 1.25 µg/mL, Abcam

[b] Goat anti-mouse IgG1-PE-Cy7, 0.5 µg/mL, eBioscience

[c] Goat anti-mouse IgG2a-AF488, 5 µg/mL, Invitrogen

[d] Goat anti-chicken IgY-Qdot655, 2.5nM, Exbio

[e] Goat anti-mouse IgG2a-PerCp-eFluor710, 0.5 µg/mL, eBioscience

Supplementay table 5: Immune cells infiltrating each tumoral lesion.

ND: not determined

cell population phenotype		total cells live cells	CD45⁻/non immune cells CD45⁻	CD45⁺ / immune cells CD45⁺		CD3⁺T cells CD3⁺		γδ T cells CD3⁺ γδTCR⁺		NKT cells CD3⁺ CD16⁺		CD4⁻ CD8⁺T cells CD3⁺ gdTCR⁻ CD4⁻ CD8⁺		CD4⁺ CD8⁺T cells CD3⁺ gdTCR⁻ CD4⁺ CD8⁺	
Pig_Lesion	Stage of regression	count/g	count/g	% of total	count/g	count/g	% of CD45⁺	count/g	% of CD45⁺	count/g	% of CD45⁺	count/g	% of CD45⁺	count/g	% of CD45⁺
1331 L3	R0	18914891	12115948	35.9	6798943	164324	2.42	52792	0.776	3352	0.049	16777	0.247	1011	0.015
1336 L2	R0	55880346	16638629	70.2	39241717	214885	0.55	110887	0.283	3572	0.009	21790	0.056	919	0.002
1343_L1	R0	29777126	13017726	56.3	16759400	249475	1.49	68325	0.408	9752	0.058	11411	0.068	1373	0.008
1376 L1	R0	13295821	6702464	49.6	6593356	182840	2.77	119928	1.819	2707	0.041	11151	0.169	4633	0.070
1377 L3	R0	54166625	34370465	36.6	19796160	810155	4.09	290564	1.468	17127	0.087	131511	0.664	23066	0.117
1378 L1	R0	40736516	25950609	36.3	14785907	339841	2.30	116471	0.788	17981	0.122	30510	0.206	8294	0.056
1387 L2	R0	33491705	20100095	40.0	13391610	400653	2.99	220737	1.648	6090	0.045	17261	0.129	12851	0.096
1396_L2	R0	24454070	17401728	28.8	7052342	209085	2.97	150999	2.141	2367	0.034	16094	0.228	5241	0.074
1399 L1	R0	15180321	11321545	25.4	3858777	85279	2.21	39932	1.035	1122	0.029	3781	0.098	2520	0.065
1472 L2	R0	42957060	31444364	26.8	11512696	508478	4.42	191475	1.663	10355	0.090	75949	0.660	3027	0.026
1474 L2	R0	61807716	36029060	41.7	25778656	646454	2.51	193247	0.750	27580	0.107	70192	0.272	11305	0.044
1475 L2	R0	64569732	42871013	33.6	21698718	1750200	8.07	542240	2.499	33894	0.156	447985	2.065	54134	0.249
1519 L3	R0	58326022	47685680	18.2	10640342	418516	3.93	ND	ND	ND	ND	ND	ND	ND	ND
1331 L8	R1	36803764	29834003	18.9	6969761	371976	5.34	185242	2.658	5598	0.080	43316	0.621	7088	0.102
1335 L2	R1	57969194	36081778	37.8	21887416	1714926	7.84	743135	3.395	60722	0.277	225939	1.032	103459	0.473
1336 L4	R1	54408237	29046261	46.6	25361976	1589895	6.27	739557	2.916	38808	0.153	204372	0.806	38966	0.154
1341 L3	R1	44879806	27448400	38.8	17431406	542894	3.11	170196	0.976	11538	0.066	106328	0.610	8875	0.051
1362 L4	R1	35984628	25154177	30.1	10830451	436603	4.03	305199	2.818	11746	0.108	25999	0.240	12530	0.116
1364 L1	R1	50282843	25696605	48.9	24586238	847580	3.45	415022	1.688	9340	0.038	144673	0.588	14486	0.059
1367 L2	R1	23447958	13542933	42.2	9905025	160423	1.62	64540	0.652	10200	0.103	16970	0.171	2643	0.027
1377 L6	R1	38482960	20740352	46.1	17742607	220494	1.24	145719	0.821	6661	0.038	14905	0.084	4996	0.028
1387_L1	R1	20517156	13713797	33.0	6803359	384517	5.65	186109	2.736	7867	0.116	22221	0.327	10626	0.156
1397 L2	R1	13934503	6294191	54.8	7640312	140325	1.84	53617	0.702	7320	0.096	7050	0.092	2952	0.039
1470 L1	R1	95387280	67144291	29.6	28242988	1660758	5.88	367146	1.300	204904	0.726	375092	1.328	34176	0.121
1473 L1	R1	54209902	39475828	27.2	14734074	606840	4.12	245433	1.666	8387	0.057	108700	0.738	5375	0.036
1516 L1	R1	18667535	16210263	13.2	2457273	48552	1.98	ND	ND	ND	ND	ND	ND	ND	ND
1516_L11	R1	34010331	24639378	27.6	9370953	227874	2.43	ND	ND	ND	ND	ND	ND	ND	ND
1516 L2	R1	46590226	35560873	23.7	11029353	ND	ND	ND	ND	ND	ND	ND	ND	ND	ND
1516 L7	R1	22257965	19650757	11.7	2607208	16395	0.63	ND	ND	ND	ND	ND	ND	ND	ND
1516 L9	R1	14209991	12470526	12.2	1739465	64116	3.69	ND	ND	ND	ND	ND	ND	ND	ND
1518 L1	R1	57233423	47265659	17.4	9967764	178374	1.79	ND	ND	ND	ND	ND	ND	ND	ND
1331_L9	R2	35017153	15866565	54.7	19150588	510961	2.67	266615	1.392	11200	0.058	37554	0.196	16823	0.088
1335 L1	R2	59834851	29054592	51.4	30780259	813010	2.64	488965	1.589	27082	0.088	43790	0.142	10779	0.035
1336 L5	R2	28233312	18695909	33.8	9537402	548609	5.75	263569	2.764	12836	0.135	60991	0.639	32051	0.336
1341 L1	R2	9373131	7717702	17.7	1655430	38369	2.32	13968	0.844	3416	0.206	3433	0.207	998	0.060
1343_L4	R2	43580208	20057491	54.0	23522718	584911	2.49	276067	1.174	17582	0.075	27527	0.117	21401	0.091
1362 L1	R2	48810600	27583274	43.5	21227326	972479	4.58	585636	2.759	11219	0.053	118065	0.556	30154	0.142
1364 L3	R2	14256656	7564475	46.9	6692181	439859	6.57	187134	2.796	17807	0.266	75982	1.135	17462	0.261
1367 L4	R2	37984121	20756489	45.4	17227632	206184	1.20	72472	0.421	23048	0.134	12564	0.073	5242	0.030
1377 L5	R2	34520139	25512036	26.1	9008104	211740	2.35	190841	2.119	719	0.008	7066	0.078	539	0.006
1378_L3	R2	29906883	21561362	27.9	8345521	133516	1.60	116335	1.394	631	0.008	1505	0.018	0	0.000
1387 L3	R2	11928037	7059912	40.8	4868125	357370	7.34	104631	2.149	8449	0.174	105984	2.177	19573	0.402
1396 L4	R2	4167625	3306469	20.7	861156	131661	15.29	66307	7.700	3281	0.381	10225	1.187	3052	0.354
1470 L3	R2	58190971	32778155	43.7	25412816	2538913	9.99	875392	3.445	19669	0.077	365254	1.437	73965	0.291
1472 L1	R2	49396750	22043651	55.4	27353099	6221757	22.75	373312	1.365	93507	0.342	3399710	12.429	131757	0.482
1473_L2	R2	44913883	28008651	37.6	16905232	4658204	27.56	418351	2.475	59421	0.351	2735242	16.180	275039	1.627
1474 L3	R2	51627459	28725840	44.4	22901619	2295296	10.02	751580	3.282	14382	0.063	384994	1.681	100527	0.439
1475 L3	R2	30372087	18737871	38.3	11634216	392769	3.38	82847	0.712	3969	0.034	40809	0.351	10062	0.086
1479 L1	R2	15991561	10910493	31.8	5081068	465020	9.15	34468	0.678	8789	0.173	190326	3.746	13192	0.260
1483 L2	R2	23584906	15745334	33.2	7839571	292044	3.73	84984	1.084	3145	0.040	24879	0.317	3319	0.042
1516 L8	R2	13445220	10923689	18.8	2521531	ND	ND	ND	ND	ND	ND	ND	ND	ND	ND
1517 L1	R2	28778495	23311341	19.0	5467154	205465	3.76	ND	ND	ND	ND	ND	ND	ND	ND
1519 L2	R2	11462430	10168591	11.3	1293840	ND	ND	ND	ND	ND	ND	ND	ND	ND	ND
1331 L4	R3	15572519	9303973	40.3	6268546	1109704	17.70	274491	4.379	22296	0.356	396899	6.332	28835	0.460
1335 L5	R3	24786325	14889368	39.9	9896956	412359	4.17	265801	2.686	6104	0.062	53866	0.544	5820	0.059
1336 L3	R3	14636364	10364173	29.2	4272191	1001474	23.44	109159	2.555	16487	0.386	610043	14.279	19853	0.465
1362 L3	R3	30818479	18098020	41.3	12720459	450530	3.54	264154	2.077	9406	0.074	60830	0.478	16869	0.133
1362 L5	R3	31969625	23881445	25.3	8088180	279149	3.45	198021	2.448	1869	0.023	14697	0.182	1274	0.016
1364 L4	R3	18435882	15074871	18.2	3361011	54172	1.61	41956	1.248	359	0.011	3012	0.090	24	0.001
1367 L6	R3	64526828	19618256	69.6	44908572	298569	0.67	208287	0.464	5450	0.012	16232	0.036	0	0.000
1377 L4	R3	56772292	20162874	64.5	36609418	5393186	14.73	483571	1.321	169383	0.463	2254401	6.158	325102	0.888
1467 L3	R3	24876634	14371457	42.2	10505177	1424794	13.56	551759	5.252	12050	0.115	199382	1.898	28647	0.273
1470 L2	R3	5320755	4151516	22.0	1169238	131525	11.25	25951	2.219	578	0.049	64094	5.482	3039	0.260
1472 L5	R3	9575758	7489001	21.8	2086756	159503	7.64	28494	1.365	1226	0.059	71302	3.417	3015	0.144
1473_L3	R3	5394737	3498411	35.2	1896326	141669	7.47	53854	2.840	3886	0.205	41110	2.168	4119	0.217
1474 L8	R3	6450000	4841695	24.9	1608305	114927	7.15	52073	3.238	2848	0.177	34512	2.146	3051	0.190
1475 L8	R3	11862745	9675661	18.4	2187084	90670	4.15	31763	1.452	638	0.029	28421	1.300	2628	0.120
1479 L3	R3	39519231	15072809	61.9	24446421	7477182	30.59	496548	2.031	264551	1.082	3834543	15.686	306110	1.252
1482 L4	R3	98965517	45642887	53.9	53322630	12412690	23.28	1712515	3.212	664037	1.245	6922955	12.983	807329	1.514
1490 L2	R3	110338983	32729930	70.3	77609053	2561697	3.30	862522	1.111	78805	0.102	590068	0.760	191107	0.246
1331 L2	R4	38509269	14231873	63.0	24277396	7176940	29.56	1351876	5.568	235689	0.971	1946359	8.017	278773	1.148
1331 L5	R4	38348752	24369352	36.5	13979400	6317726	45.19	1167629	8.352	277654	1.986	2250458	16.098	943313	6.748
1331 L6	R4	33403974	20015619	40.1	13388356	1347475	10.07	255360	1.907	44937	0.336	508355	3.797	31233	0.233
1331 L7	R4	46217365	20313748	56.1	25903617	1716742	6.63	1314195	5.073	19227	0.074	80488	0.311	24835	0.096
1335 L3	R4	20095696	9291223	53.8	10804472	2013634	18.64	227873	2.109	58704	0.543	1216588	11.260	100927	0.934
1335 L6	R4	43690828	22392949	48.8	21297879	7598704	35.68	686826	3.225	319144	1.498	4497569	21.117	523252	2.457
1336 L1	R4	25758323	16317860	36.7	9440463	5163675	54.70	1449641	15.356	316445	3.352	1359812	14.404	747178	7.915
1336 L7	R4	33720216	26632055	21.0	7088162	1521242	21.46	1196070	16.874	6615	0.093	50134	0.707	63189	0.891
1364_L2	R4	27773601	12334870	55.6	15438730	7153012	46.33	677246	4.387	114552	0.742	4289413	27.783	147106	0.953
1364 L6	R4	26051966	13634441	47.7	12417525	3072981	24.75	681686	5.490	30955	0.249	864444	6.961	410343	3.305
1367 L5	R4	38010913	21901812	42.4	16109100	3090091	19.18	303413	1.883	86179	0.535	1540537	9.563	150427	0.934
1479 L4	R4	17772727	14022486	21.1	3750241	405893	10.82	108708	2.899	4174	0.111	110656	2.951	38771	1.034
1480 L1	R4	47209302	23651658	49.9	23557644	11037850	46.86	1424035	6.045	168325	0.715	6579890	27.931	628603	2.668
1483_L1	R4	40689655	28070249	31.0	12619406	2616401	20.73	1290438	10.226	37190	0.295	312298	2.475	86209	0.683

cell population phenotype	CD4⁺ CD8⁻T cells CD3⁺ gdTCR⁻ CD4⁺ CD8⁻		CD4⁻ CD8⁻T cells CD3⁺ gdTCR⁻ CD4⁻ CD8⁻		NK cells CD3⁻ CD8⁺ CD16⁺		B cells CD79a⁺		DC CD172a⁺ MHCII^{hi} FSC^{hi}		DC CD172a⁺ CD163^{low} CD172a⁺ MHCII^{hi} FSC^{hi}		DC CD172a⁺ CD163^{hi} CD172a⁺ MHCII^{hi} FSC^{hi}		DC CD172a⁺ CD163⁻ CD172a⁺ MHCII^{hi} FSC^{hi}		DC CD172a^{low} CD163⁺ CD172a^{low} MHCII^{hi} FSC^{hi}	
Pig_Lesion	count/g	% of CD45⁺	count/g	% of CD45⁺	count/g	% of CD45⁺	count/g	% of CD45⁺	count/g	% of CD45⁺	count/g	% of CD45⁺	count/g	% of CD45⁺	count/g	% of CD45⁺	count/g	% of CD45⁺
1331 L3	74904	1.102	14656	0.216	8349	0.123	23160	0.341	39390	0.579	7624	0.112	2308	0.034	12373	0.182	17746	0.261
1336 L2	64195	0.164	12808	0.033	4082	0.010	16336	0.042	102981	0.262	32853	0.084	13162	0.034	33590	0.086	32853	0.084
1343_L1	137793	0.822	18904	0.113	2145	0.013	22821	0.136	101157	0.604	14651	0.087	4884	0.029	34411	0.205	39688	0.237
1376 L1	31446	0.477	12975	0.197	21278	0.323	7019	0.106	235742	3.575	62312	0.945	36838	0.559	110439	1.675	62221	0.944
1377 L3	127897	0.646	219214	1.107	52415	0.265	78157	0.395	554798	2.803	405699	2.049	306579	1.549	75859	0.383	71039	0.359
1378 L1	98316	0.665	67110	0.454	45417	0.307	13612	0.092	437407	2.958	175953	1.190	95467	0.646	123205	0.833	136726	0.925
1387 L2	131493	0.982	13943	0.104	27046	0.202	13506	0.101	453440	3.386	229169	1.711	128031	0.956	121548	0.908	97008	0.724
1396_L2	40776	0.578	12070	0.171	14741	0.209	6189	0.088	165081	2.341	62660	0.889	27245	0.386	54933	0.779	46361	0.657
1399 L1	29791	0.772	8585	0.222	5100	0.132	4625	0.120	114955	2.979	48583	1.259	25062	0.649	27454	0.711	37505	0.972
1472 L2	105566	0.917	122282	1.062	17331	0.151	5884	0.051	114295	0.993	63271	0.550	24067	0.209	13904	0.121	34187	0.297
1474 L2	212565	0.825	132123	0.513	21927	0.085	53919	0.209	419203	1.626	331530	1.286	237428	0.921	28111	0.109	56789	0.220
1475 L2	492418	2.269	176775	0.815	77309	0.356	7230	0.033	637568	2.938	369694	1.704	185058	0.853	99355	0.458	160159	0.738
1519 L3	ND	ND	ND	ND	11631	0.109	ND	ND	510253	4.795	295309	2.775	238569	2.242	123108	1.157	87944	0.827
1331 L8	94112	1.350	34841	0.500	12346	0.177	7243	0.104	97837	1.404	43167	0.619	23552	0.338	33646	0.483	19830	0.285
1335 L2	517640	2.365	53113	0.243	76038	0.347	36338	0.166	152923	0.699	56388	0.258	37880	0.173	43610	0.199	49175	0.225
1336 L4	403946	1.593	155494	0.613	51726	0.204	38595	0.152	431785	1.702	109842	0.433	64939	0.256	208447	0.822	111557	0.440
1341 L3	194408	1.115	48780	0.280	35395	0.203	7599	0.044	454751	2.609	91668	0.526	54136	0.311	214026	1.228	148597	0.852
1362 L4	57427	0.530	23597	0.218	83687	0.773	4885	0.045	421901	3.896	162801	1.503	58290	0.538	139265	1.286	109469	1.011
1364 L1	121546	0.494	141878	0.577	36025	0.147	21600	0.088	293155	1.192	149314	0.607	105768	0.430	56908	0.231	83391	0.339
1367 L2	45299	0.457	19891	0.201	24620	0.249	9653	0.097	270904	2.735	71841	0.725	33157	0.335	96204	0.971	101730	1.027
1377 L6	34223	0.193	13906	0.078	16903	0.095	7419	0.042	376616	2.123	184452	1.040	129991	0.733	70760	0.399	118463	0.668
1387_L1	85980	1.264	71449	1.050	23924	0.352	10211	0.150	231319	3.400	73491	1.080	9598	0.141	38041	0.559	116875	1.718
1397 L2	48490	0.635	21740	0.285	10238	0.134	10970	0.144	150488	1.970	51164	0.670	8293	0.109	44142	0.578	53053	0.694
1470 L1	382344	1.354	300413	1.064	186080	0.659	23695	0.084	719801	2.549	323228	1.144	120457	0.427	135834	0.481	250939	0.889
1473 L1	76359	0.518	162819	1.105	13947	0.095	4592	0.031	221624	1.504	142194	0.965	94217	0.639	21172	0.144	56756	0.385
1516 L1	ND	ND	ND	ND	7400	0.301	ND	ND	218402	8.888	28079	1.143	14494	0.590	56116	2.284	133142	5.418
1516_L11	ND	ND	ND	ND	30411	0.325	ND	ND	353410	3.771	62100	0.663	26785	0.286	118776	1.267	170327	1.818
1516 L2	ND	ND	ND	ND	50378	0.457	ND	ND	ND	ND	ND	ND	ND	ND	ND	ND	ND	ND
1516 L7	ND	ND	ND	ND	1261	0.048	ND	ND	164807	6.321	10019	0.384	4325	0.166	39967	1.533	114009	4.373
1516 L9	ND	ND	ND	ND	3360	0.193	ND	ND	109685	6.306	20420	1.174	12010	0.690	28717	1.651	59348	3.412
1518 L1	ND	ND	ND	ND	16874	0.169	ND	ND	175955	1.765	61390	0.616	44091	0.442	50011	0.502	63381	0.636
1331_L9	139896	0.731	39531	0.206	49677	0.259	38319	0.200	417861	2.182	163262	0.853	32029	0.167	153984	0.804	99427	0.519
1335 L1	193349	0.628	50257	0.163	79765	0.259	43250	0.141	812721	2.640	365014	1.186	83915	0.273	314937	1.023	131411	0.427
1336 L5	132329	1.387	47999	0.503	69704	0.731	29335	0.308	408456	4.283	185011	1.940	48363	0.507	122282	1.282	99732	1.046
1341 L1	11651	0.704	5039	0.304	7846	0.474	2297	0.139	101174	6.112	13981	0.845	3097	0.187	33472	2.022	53342	3.222
1343_L4	212579	0.904	31028	0.132	49565	0.211	34793	0.148	912312	3.878	214403	0.911	75286	0.320	408599	1.737	287612	1.223
1362 L1	147388	0.694	78948	0.372	170242	0.802	71987	0.339	788574	3.715	425651	2.005	249625	1.176	188990	0.890	166846	0.786
1364 L3	76656	1.145	64784	0.968	26243	0.392	8702	0.130	58583	0.875	14970	0.224	8415	0.126	10686	0.160	31921	0.477
1367 L4	73138	0.425	19720	0.114	28956	0.168	6850	0.040	558459	3.242	199249	1.157	117393	0.681	176598	1.025	180894	1.050
1377 L5	4790	0.053	7665	0.085	10958	0.122	4465	0.050	513942	5.705	268085	2.976	118778	1.319	167915	1.864	70420	0.782
1378_L3	4222	0.051	10823	0.130	22131	0.265	5319	0.064	661841	7.930	227405	2.725	95154	1.140	258153	3.093	169086	2.026
1387 L3	94469	1.941	24624	0.506	22159	0.455	4240	0.087	349876	7.187	193715	3.979	70224	1.443	52823	1.085	101829	2.092
1396 L4	28957	3.363	20068	2.330	10225	1.187	2258	0.262	63646	7.391	15662	1.819	5619	0.653	16181	1.879	31684	3.679
1470 L3	866111	3.408	338383	1.332	143221	0.564	56651	0.223	1079950	4.250	562908	2.215	410080	1.614	226432	0.891	289884	1.141
1472 L1	1123954	4.109	1102820	4.032	114586	0.419	23282	0.085	21783	0.080	9345	0.034	2475	0.009	2166	0.008	9468	0.035
1473_L2	567144	3.355	606133	3.585	65527	0.388	20030	0.118	27086	0.160	10511	0.062	3003	0.018	3465	0.020	12301	0.073
1474 L3	563757	2.462	480200	2.097	117929	0.515	29381	0.128	685776	2.994	441354	1.927	258175	1.127	53542	0.234	188130	0.821
1475 L3	142048	1.221	113146	0.973	23255	0.200	11377	0.098	146615	1.260	59868	0.515	28532	0.245	39601	0.340	46788	0.402
1479 L1	89350	1.758	128926	2.537	8805	0.173	12718	0.250	137420	2.705	33803	0.665	12621	0.248	45795	0.901	57567	1.133
1483 L2	67588	0.862	108128	1.379	10216	0.130	24021	0.306	256442	3.271	47108	0.601	14177	0.181	87314	1.114	120870	1.542
1516 L8	ND	ND	ND	ND	ND	ND	ND	ND	ND	ND	ND	ND	ND	ND	ND	ND	ND	ND
1517 L1	ND	ND	ND	ND	40882	0.748	ND	ND	83020	1.519	20184	0.369	13456	0.246	16756	0.306	44303	0.810
1519 L2	ND	ND	ND	ND	ND	ND	ND	ND	ND	ND	ND	ND	ND	ND	ND	ND	ND	ND
1331 L4	180149	2.874	205366	3.276	35645	0.569	13498	0.215	430207	6.863	313229	4.997	227502	3.629	48789	0.778	63952	1.020
1335 L5	55044	0.556	25396	0.257	103918	1.050	9795	0.099	324068	3.274	131109	1.325	65805	0.665	93718	0.947	91646	0.926
1336 L3	99862	2.337	144775	3.389	30243	0.708	2331	0.055	104314	2.442	53346	1.249	34003	0.796	23349	0.547	26404	0.618
1362 L3	65727	0.517	33427	0.263	70547	0.555	18362	0.144	598955	4.709	245757	1.932	140701	1.106	150896	1.186	193627	1.522
1362 L5	34065	0.421	29053	0.359	41796	0.517	5215	0.064	569132	7.037	347855	4.301	171063	2.115	118660	1.467	96887	1.198
1364 L4	2486	0.074	6311	0.188	16089	0.479	4342	0.129	115638	3.441	54022	1.607	24911	0.741	38070	1.133	22718	0.676
1367 L6	11729	0.026	56278	0.125	17180	0.038	20120	0.045	801075	1.784	297750	0.663	146414	0.326	322050	0.717	178301	0.397
1377 L4	1451486	3.965	697212	1.904	97711	0.267	145016	0.396	284567	0.777	177022	0.484	120053	0.328	49299	0.135	51217	0.140
1467 L3	310844	2.959	322531	3.070	128477	1.223	47242	0.450	284776	2.711	90045	0.857	37804	0.360	89156	0.849	103824	0.988
1470 L2	21197	1.813	16667	1.425	8986	0.769	4293	0.367	64637	5.528	13758	1.177	5797	0.496	19506	1.668	31224	2.670
1472 L5	23226	1.113	32305	1.548	14181	0.680	3831	0.184	105218	5.042	8421	0.404	3260	0.156	28221	1.352	67821	3.250
1473_L3	18185	0.959	20438	1.078	12356	0.652	33875	1.786	100136	5.281	28332	1.494	17591	0.928	29178	1.539	42118	2.221
1474 L8	7730	0.481	14578	0.906	15798	0.982	16790	1.044	139390	8.667	18049	1.122	7967	0.495	39272	2.442	81434	5.063
1475 L8	15093	0.690	12127	0.554	8260	0.378	4569	0.209	138146	6.316	23995	1.097	8697	0.398	45855	2.097	68102	3.114
1479 L3	1238171	5.065	1341049	5.486	86214	0.353	27177	0.111	214810	0.879	65980	0.270	17560	0.072	21864	0.089	122964	0.503
1482 L4	1334064	2.502	979079	1.836	352089	0.660	95009	0.178	144979	0.272	46862	0.088	7581	0.014	45484	0.085	51600	0.097
1490 L2	624611	0.805	217425	0.280	136078	0.175	118791	0.153	1569120	2.022	1079751	1.391	633653	0.816	322447	0.415	164611	0.212
1331 L2	1363702	5.617	1980640	8.158	85804	0.353	93611	0.386	46260	0.191	16608	0.068	10810	0.045	15279	0.063	13830	0.057
1331 L5	980747	7.016	697236	4.988	134119	0.959	21569	0.154	50944	0.364	6223	0.045	2268	0.016	11391	0.081	32697	0.234
1331 L6	328330	2.452	175851	1.313	73387	0.548	17354	0.130	375091	2.802	141393	1.056	64972	0.485	86868	0.649	141321	1.056
1331 L7	179189	0.692	98647	0.381	170109	0.657	8203	0.032	860592	3.322	633135	2.444	344993	1.332	131070	0.506	89935	0.347
1335 L3	350652	3.245	53473	0.495	55278	0.512	23429	0.217	448125	4.148	300912	2.785	140528	1.301	44458	0.411	97006	0.898
1335 L6	1187913	5.578	383896	1.803	245566	1.153	23711	0.111	915805	4.300	474444	2.228	251461	1.181	110401	0.518	317266	1.490
1336 L1	644058	6.822	643178	6.813	102920	1.090	21345	0.226	113498	1.202	42713	0.452	22292	0.236	24494	0.259	44420	0.471
1336 L7	164675	2.323	38819	0.548	48915	0.690	6124	0.086	109971	1.551	76647	1.081	51542	0.727	17773	0.251	12663	0.179
1364_L2	843069	5.461	1072172	6.945	125073	0.810	37296	0.242	38092	0.247	19769	0.128	13434	0.087	4847	0.031	11819	0.077
1364 L6	685637	5.522	401520	3.233	73614	0.593	72659	0.585	141173	1.137	82520	0.665	49101	0.395	28876	0.233	27507	0.222
1367 L5	710021	4.408	299108	1.857	155057	0.963	100916	0.626	358065	2.223	201008	1.248	129789	0.806	92073	0.572	53010	0.329
1479 L4	76801	2.048	66690	1.778	29681	0.791	61598	1.642	94255	2.513	51303	1.368	27658	0.738	28092	0.749	14209	0.379
1480 L1	1402600	5.954	836226	3.550	201558	0.856	245460	1.042	869779	3.692	345412	1.466	102282	0.434	191760	0.814	300673	1.276
1483_L1	674297	5.343	216482	1.715	74893	0.593	144233	1.143	326776	2.589	170598	1.352	80553	0.638	78112	0.619	70246	0.557

cell population phenotype	PMN CD172a⁺ PG68A⁺		macrophages CD172a⁺ PG68A⁻ MHCII$^{-/int}$		MHCII⁻ CD14⁻ macrophages CD172a⁺ PG68A⁻ MHCII⁻ CD14⁻			MHCII⁻ CD14⁺ macrophages CD172a⁺ PG68A⁻ MHCII⁻ CD14⁺			MHCII⁺ CD163⁺ macrophages CD172a⁺ PG68A⁻ MHCII⁺ CD163⁺			MHCII⁺ CD163⁻ macrophages CD172a⁺ PG68A⁻ MHCII⁺ CD163⁻		
Pig_Lesion	count/g	% of CD45⁺	count/g	% of CD45⁺	count/g	% of CD45⁺	% of macrophages	count/g	% of CD45⁺	% of macrophages	count/g	% of CD45⁺	% of macrophages	count/g	% of CD45⁺	% of macrophages
1331 L3	1044492	15.36	5481730	80.63	1844529	27.1	33.6	3292020	48.4	60.1	201490	3.0	3.7	144013	2.1	2.6
1336 L2	27188994	69.29	10698486	27.26	6785508	17.3	63.4	2600334	6.6	24.3	930834	2.4	8.7	381811	1.0	3.6
1343_L1	3020790	18.02	13148396	78.45	5044997	30.1	38.4	6180909	36.9	47.0	1096897	6.5	8.3	825592	4.9	6.3
1376 L1	891249	13.52	5187151	78.67	1540104	23.4	29.7	1637219	24.8	31.6	1107253	16.8	21.3	902575	13.7	17.4
1377 L3	7121421	35.97	10167411	51.36	3948021	19.9	38.8	2541172	12.8	25.0	2954100	14.9	29.1	727576	3.7	7.2
1378 L1	1620775	10.96	11497131	77.76	3882713	26.3	33.8	5781446	39.1	50.3	911948	6.2	7.9	923945	6.2	8.0
1387 L2	4224504	31.55	7791082	58.18	2925029	21.8	37.5	1899771	14.2	24.4	2187337	16.3	28.1	779569	5.8	10.0
1396_L2	1853645	26.28	4393613	62.30	1629606	23.1	37.1	1718907	24.4	39.1	764598	10.8	17.4	193051	2.7	4.4
1399 L1	621193	16.10	2810981	72.85	905900	23.5	32.2	1470530	38.1	52.3	235851	6.1	8.4	145669	3.8	5.2
1472 L2	169359	1.47	10434800	90.64	5859546	50.9	56.2	4158851	36.1	39.9	257717	2.2	2.5	158814	1.4	1.5
1474 L2	2716402	10.54	20875417	80.98	14053528	54.5	67.3	3363453	13.0	16.1	3252145	12.6	15.6	206481	0.8	1.0
1475 L2	671969	3.10	17950774	82.73	10336165	47.6	57.6	4870319	22.4	27.1	2044175	9.4	11.4	700116	3.2	3.9
1519 L3	766370	7.20	8653407	81.33	2488175	23.4	28.8	940073	8.8	10.9	4476407	42.1	51.7	757835	7.1	8.8
1331 L8	1424453	20.44	4567538	65.53	2031374	29.1	44.5	2021233	29.0	44.3	305751	4.4	6.7	209179	3.0	4.6
1335 L2	3602603	16.46	15613435	71.34	8122663	37.1	52.0	4976976	22.7	31.9	1593742	7.3	10.2	924754	4.2	5.9
1336 L4	5590646	22.04	16363439	64.52	8171114	32.2	49.9	6100593	24.1	37.3	889429	3.5	5.4	1209342	4.8	7.4
1341 L3	3185191	18.27	12408645	71.19	4336431	24.9	34.9	6221355	35.7	50.1	1141205	6.5	9.2	711527	4.1	5.7
1362 L4	437775	4.04	8357087	77.16	2738072	25.3	32.8	3266337	30.2	39.1	1235555	11.4	14.8	1117473	10.3	13.4
1364 L1	5177787	21.06	15668831	63.73	5712099	23.2	36.5	8645744	35.2	55.2	486499	2.0	3.1	829077	3.4	5.3
1367 L2	1348876	13.62	7467601	75.39	2354887	23.8	31.5	2690977	27.2	36.0	1164430	11.8	15.6	1258198	12.7	16.8
1377 L6	6341012	35.74	10033629	56.55	2958784	16.7	29.5	4072651	23.0	40.6	1911463	10.8	19.1	1090731	6.1	10.9
1387_L1	993774	14.61	4196721	61.69	1378313	20.3	32.8	1288218	18.9	30.7	725662	10.7	17.3	804848	11.8	19.2
1397 L2	2607287	34.13	4284876	56.08	2254733	29.5	52.6	848246	11.1	19.8	704248	9.2	16.4	477871	6.3	11.2
1470 L1	1570462	5.56	23253074	82.33	13922642	49.3	59.9	5712806	20.2	24.6	2648770	9.4	11.4	969911	3.4	4.2
1473 L1	887200	6.02	12258514	83.20	7178063	48.7	58.6	3564292	24.2	29.1	1258482	8.5	10.3	257724	1.7	2.1
1516 L1	310163	12.62	1631789	66.41	708709	28.8	43.4	310589	12.6	19.0	311753	12.7	19.1	303704	12.4	18.6
1516_L11	2501963	26.70	5868840	62.63	3165201	33.8	53.9	911002	9.7	15.5	990685	10.6	16.9	811193	8.7	13.8
1516 L2	ND	ND	ND	ND	ND	ND	ND	ND	ND	ND	ND	ND	ND	ND	ND	ND
1516 L7	466129	17.88	1632136	62.60	773272	29.7	47.4	459317	17.6	28.1	125992	4.8	7.7	276329	10.6	16.9
1516 L9	219019	12.59	1230137	70.72	630842	36.3	51.3	219608	12.6	17.9	146677	8.4	11.9	235806	13.6	19.2
1518 L1	2563755	25.72	6436918	64.58	2313855	23.2	35.9	1728834	17.3	26.9	1527669	15.3	23.7	875746	8.8	13.6
1331_L9	4908043	25.63	11903872	62.16	6315018	33.0	53.1	1749087	9.1	14.7	1781784	9.3	15.0	2064997	10.8	17.3
1335 L1	9034584	29.35	17945163	58.30	8893524	28.9	49.6	2864059	9.3	16.0	4256645	13.8	23.7	1942214	6.3	10.8
1336 L5	915184	9.60	6961923	73.00	2028028	21.3	29.1	1713094	18.0	24.6	1886887	19.8	27.1	1339752	14.0	19.2
1341 L1	142403	8.60	1203038	72.67	365229	22.1	30.4	447230	27.0	37.2	133022	8.0	11.1	258286	15.6	21.5
1343_L4	6200741	26.36	14377183	61.12	6207099	26.4	43.2	3600528	15.3	25.0	2580888	11.0	18.0	1999622	8.5	13.9
1362 L1	3646622	17.18	11539853	54.36	3185540	15.0	27.6	2698932	12.7	23.4	4006566	18.9	34.7	1650748	7.8	14.3
1364 L3	520085	7.77	5249322	78.44	1459581	21.8	27.8	1939513	29.0	36.9	928597	13.9	17.7	921722	13.8	17.6
1367 L4	1063883	6.18	12251413	71.12	2300928	13.4	18.8	5476879	31.8	44.7	1592115	9.2	13.0	2884225	16.7	23.5
1377 L5	205187	2.28	6913951	76.75	1286164	14.3	18.6	1912309	21.2	27.7	1967854	21.8	28.5	1747625	19.4	25.3
1378_L3	1601311	19.19	4785411	57.34	1116017	13.4	23.3	1899526	22.8	39.7	820131	9.8	17.1	949737	11.4	19.8
1387 L3	123136	2.53	3313081	68.06	1108089	22.8	33.4	747693	15.4	22.6	710006	14.6	21.4	747515	15.4	22.6
1396 L4	24151	2.81	574051	66.66	225452	26.2	39.3	191456	22.2	33.4	31086	3.6	5.4	126057	14.6	22.0
1470 L3	2252902	8.87	17281556	68.00	5412440	21.3	31.3	6096451	24.0	35.3	2787892	11.0	16.1	2987674	11.8	17.3
1472 L1	832537	3.04	16952981	61.98	4278408	15.6	25.2	3027221	11.1	17.9	7832640	28.6	46.2	1817186	6.6	10.7
1473_L2	702144	4.15	10273496	60.77	3491953	20.7	34.0	1075451	6.4	10.5	4379422	25.9	42.6	1328288	7.9	12.9
1474 L3	647270	2.83	15627625	68.24	5782889	25.3	37.0	5080794	22.2	32.5	3310795	14.5	21.2	1454432	6.4	9.3
1475 L3	2743001	23.58	7717646	66.34	3243434	27.9	42.0	2410457	20.7	31.2	1429572	12.3	18.5	635261	5.5	8.2
1479 L1	349817	6.89	3822139	75.22	1020440	20.1	26.7	665170	13.1	17.4	1378547	27.1	36.1	758612	14.9	19.8
1483 L2	1051974	13.42	5338458	68.10	1209350	15.4	22.7	2095071	26.7	39.2	1385829	17.7	26.0	649107	8.3	12.2
1516 L8	ND	ND	ND	ND	ND	ND	ND	ND	ND	ND	ND	ND	ND	ND	ND	ND
1517 L1	1282879	23.47	3402055	62.23	2155735	39.4	63.4	550295	10.1	16.2	517925	9.5	15.2	179116	3.3	5.3
1519 L2	ND	ND	ND	ND	ND	ND	ND	ND	ND	ND	ND	ND	ND	ND	ND	ND
1331 L4	621853	9.92	3572424	56.99	970648	15.5	27.2	557056	8.9	15.6	1246834	19.9	34.9	798217	12.7	22.3
1335 L5	1359963	13.74	6784772	68.55	3252814	32.9	47.9	1057572	10.7	15.6	1120386	11.3	16.5	1354167	13.7	20.0
1336 L3	50755	1.19	2549358	59.67	494402	11.6	19.4	741879	17.4	29.1	858011	20.1	33.7	455253	10.7	17.9
1362 L3	1472950	11.58	8779350	69.02	1955461	15.4	22.3	3026011	23.8	34.5	1715742	13.5	19.5	2082389	16.4	23.7
1362 L5	69279	0.86	5008219	61.92	1488724	18.4	29.7	862502	10.7	17.2	1404235	17.4	28.0	1252758	15.5	25.0
1364 L4	46036	1.37	2722716	81.01	668730	19.9	24.6	846336	25.2	31.1	586673	17.5	21.5	620977	18.5	22.8
1367 L6	23002006	51.22	16200818	36.08	4466250	9.9	27.6	2358930	5.3	14.6	4468916	10.0	27.6	4906723	10.9	30.3
1377 L4	1390610	3.80	26341617	71.95	4321281	11.8	16.4	3135629	8.6	11.9	12886880	35.2	48.9	5997828	16.4	22.8
1467 L3	452724	4.31	7598348	72.33	2003131	19.1	26.4	2630607	25.0	34.6	1995036	19.0	26.3	970407	9.2	12.8
1470 L2	22665	1.94	788308	67.42	341521	29.2	43.3	185552	15.9	23.5	84192	7.2	10.7	177441	15.2	22.5
1472 L5	84874	4.07	1540295	73.81	557086	26.7	36.2	299203	14.3	19.4	260750	12.5	16.9	423527	20.3	27.5
1473_L3	41780	2.20	1303798	68.75	609950	32.2	46.8	207715	11.0	15.9	347600	18.3	26.7	139040	7.3	10.7
1474 L8	18684	1.16	1127527	70.11	674457	41.9	59.8	151517	9.4	13.4	134243	8.3	11.9	167663	10.4	14.9
1475 L8	37235	1.70	1719569	78.62	556585	25.4	32.4	549673	25.1	32.0	138030	6.3	8.0	475980	21.8	27.7
1479 L3	137425	0.56	14972831	61.25	1202566	4.9	8.0	1435539	5.9	9.6	8026302	32.8	53.6	4311824	17.6	28.8
1482 L4	588272	1.10	38118359	71.49	5451830	10.2	14.3	4201548	7.9	11.0	20348226	38.2	53.4	8124594	15.2	21.3
1490 L2	21984766	28.33	49225236	63.43	18395809	23.7	37.4	3141473	4.0	6.4	18632332	24.0	37.9	9063629	11.7	18.4
1331 L2	1607260	6.62	14356517	59.14	1381939	5.7	9.6	908289	3.7	6.3	8213309	33.8	57.2	3857268	15.9	26.9
1331 L5	90075	0.64	6548714	46.85	3242899	23.2	49.5	977426	7.0	14.9	1480642	10.6	22.6	848432	6.1	13.0
1331 L6	269762	2.02	9417988	70.35	1806833	13.5	19.2	2692254	20.1	28.6	2961801	22.1	31.4	1957099	14.6	20.8
1331 L7	903910	3.49	12613169	48.69	3964757	15.3	31.4	987976	3.8	7.8	4171355	16.1	33.1	3489081	13.5	27.7
1335 L3	92460	0.86	7193198	66.58	1078829	10.0	15.0	1515054	14.0	21.1	2324795	21.5	32.3	2275724	21.1	31.6
1335 L6	417971	1.96	10232725	48.05	2205348	10.4	21.6	1875721	8.8	18.3	3875415	18.2	37.9	2278423	10.7	22.3
1336 L1	70070	0.74	3731854	39.53	912556	9.7	24.5	315176	3.3	8.4	1467003	15.5	39.3	1038549	11.0	27.8
1336 L7	25327	0.36	4665235	65.82	1911504	27.0	41.0	181508	2.6	3.9	2015033	28.4	43.2	558522	7.9	12.0
1364_L2	349166	2.26	6828360	44.23	751985	4.9	11.0	694379	4.5	10.2	3264978	21.1	47.8	2117019	13.7	31.0
1364 L6	238722	1.92	7985113	64.31	2822346	22.7	35.3	843213	6.8	10.6	2636459	21.2	33.0	1684029	13.6	21.1
1367 L5	1579449	9.81	9364845	58.13	2083180	12.9	22.2	2421915	15.0	25.9	2456358	15.2	26.2	2403393	14.9	25.7
1479 L4	187100	4.99	2760835	73.62	1760583	46.9	63.8	228967	6.1	8.3	328211	8.8	11.9	443074	11.8	16.0
1480 L1	992258	4.21	9749263	41.39	1207492	5.1	12.4	763533	3.2	7.8	2970143	12.6	30.5	4811982	20.4	49.4
1483_L1	1782373	14.12	6974985	55.27	1647033	13.1	23.6	1749781	13.9	25.1	1998084	15.8	28.6	1583116	12.5	22.7

Supplementay table 6: List of primers used for qRT-PCR.

gene	name	TaqMan assayID or SYBRGreen sequence primers	T°	efficiency (%)	product length
IL10	Interleukin 10	F: GAGCCAACTGCAGCTTCCA<br>R: TCAGGACAAATAGCCCACTAGCTT	62	95	65
IL1B	Interleukin 1 Beta	F: TGCCAACGTGCAGTCTATGG<br>R: TGGGCCAGCCAGCACTAG	60	99	70
CXCL8	C-X-C Motif Chemokine Ligand 8	F: CCGTGTCAACATGACTTCCAA<br>R: GAGCTGCAGAAAGCAGGAAAA	60	101	65
MITF-M	M isoform of Melanocyte Inducing Transcription Factor	F: GCGTGGTTATGCTGGAAATGC<br>R: TGGTTTACCTGCTGCCTTTGG	60	113	109
MLANA	Melan-A	F: CAACTGTGGACCTGTGGTTCC<br>R: CAGAAGACCTGCTGGCTCTCAT	60	118	103
TGFB1	Transforming Growth Factor Beta 1	F: AGGGCTACCATGCCAATTT<br>R: CGGGTTGTGCTGGTTGTAC	58	95	101
TNFA	Tumor Necrosis Factor	F: TGGTGGTGCCGACAGATG<br>R: CAGCCTTGGCCCCTGAA	60	109	64
TYR	Tyrosinase	F: GCCTTGGCATCGACTCTTCTT<br>R: CACAATTTTCTGCATCTCGCC	58	88	110
VEGFA	Vascular Endothelial Growth Factor A	F: CAACGACGAAGGTCTGGAGTGT<br>R: TCATCTCTCCTATGTGCTGGCC	60	123	101
HPRT1	Hypoxanthine Phosphoribosyltransferase 1	F: GGGACTTGAATCATGTTTGTGTCA<br>R: ATGGGACTCCAGATGTTTCCAA	60	107	101
RPL32	Ribosomal Protein L32	F: TGCTCTCAGACCCCTTGTGAAG<br>R: TTTCCGCCAGTTCCGCTTA	58	105	106
ARG1	Arginase 1	Ss03391394_m1	60		
CCL2	C-C Motif Chemokine Ligand 2	Ss03394377_m1	60		
CCL5	C-C Motif Chemokine Ligand 5	Ss03648939_m1	60		
CCR2	C-C Motif Chemokine Receptor 2	Ss03378154_u1	60		
CD80	CD80 Molecule	Ss03382507_u1	60		
CD86	CD86 Molecule	Ss03394401_m1	60		
CSF1	Macrophage Colony Stimulating Factor 1	Ss03373560_g1	60		
FCGR2B	Fc Fragment Of IgG Receptor Iib	Ss03392060_m1	60		
AIF1	Allograft Inflammatory Factor 1	Ss03373370_m1	60		
IDO1	Indoleamine 2,3-Dioxygenase 1	Ss04322730_m1	60		
IL4I1	Interleukin 4 Induced 1	Ss06927539_m1	60		
IL6	Interleukin 6	Ss03384604_u1	60		
LYZ	Lysozyme	Ss03394856_m1	60		
MMP2	Matrix Metallopeptidase 2	Ss03394318_m1	60		
MMP9	Matrix Metallopeptidase 9	Ss03392100_m1	60		
MRC1	Mannose Receptor C-Type 1	Ss03373693_m1	60		
MMP14	Matrix Metallopeptidase 14	Ss03394425_g1	60		
NOS2	Nitric Oxide Synthase 2	Ss03374608_u1	60		
CD274	CD274 Molecule	Ss03391948_m1	60		
S100A8	S100 Calcium Binding Protein A8	Ss04246257_g1	60		
SOCS3	Suppressor Of Cytokine Signaling 3	Ss03387992_u1	60		
HPRT1	Hypoxanthine Phosphoribosyltransferase 1	Ss03388274_m1	60		
RPL32	Ribosomal Protein L32	Ss03391553_g1	60		
MPEG1	Macrophage Expressed 1	Ss04954218_s1	60		

Supplementay table 7: Statistical description of groups by cytokines performed using v test after PCA analysis.

	R0		R1		R4	
	**v-test**	***p*-value**	**v-test**	***p*-value**	**v-test**	***p*-value**
IL-8	2.048	0.041			-2.542	0.011
TGFβ			2.247	0.025	-2.628	0.009
IL-6					-2.013	0.044
TNFα					-2.359	0.018
IL-2					-1.986	0.047
IL-1β					-2.594	0.010
IL-10					-2.628	0.009
CCL2					-2.733	0.006

Supplementay table 8: Effect of regression stages on the different proportions of cell populations among immune cells in the tumors. A linear mixed model with sex (two levels) and regression stages (5 levels) as fixed effects was used and pairwise and when a significant effect of regression stages was found, comparisons with Tukey's adjustment were calculated within regression stages. Only significant F and *p* -values are reported in the Table.

		**effect of regression stage**		**significant *p* -values of the post hoc test comparing regression stages**									
**Cell subset**		**F value**	***p* -value**	**R0 - R1**	**R0 - R2**	**R0 - R3**	**R0 - R4**	**R1 - R2**	**R1 - R3**	**R1 - R4**	**R2 - R3**	**R2 - R4**	**R3 - R4**
$CD45^-$ cells	count/g	3.08	**2.06E-02**						0.023				
CD3+ T cells	% of $CD45^+$	21.29	**9.21E-12**				<0.001			<0.001		<0.001	<0.001
γδ T cells	% of $CD45^+$	10.18	**1.62E-06**				<0.001			<0.001		<0.001	<0.001
$CD4^-$ $CD8^+$ T cells	% of $CD45^+$	9.68	**2.95E-06**				<0.001			<0.001		<0.001	0.009
$CD4^+$ $CD8^+$ T cells	% of $CD45^+$	9.06	**6.33E-06**				<0.001			<0.001		<0.001	<0.001
$CD4^+$ $CD8^-$ T cells	% of $CD45^+$	17.64	**5.67E-10**				<0.001			<0.001		<0.001	<0.001
$CD4^-$ $CD8^-$ T cells	% of $CD45^+$	7.23	**6.53E-05**				<0.001			<0.001		0.003	0.039
NKT cells	% of $CD45^+$	6.48	**1.77E-04**				<0.001			0.003		0.001	0.008
NK cells	% of $CD45^+$	14.09	**1.19E-08**		0.0358	<0.001	<0.001		0.002	<0.001		0.001	
B cells	% of $CD45^+$	3.68	**8.99E-03**							0.021		0.043	
PMN	% of $CD45^+$	5.29	**8.25E-04**			0.034	0.003			0.007			
macrophages	% of $CD45^+$	3.94	**5.89E-03**				0.005			0.020		0.047	0.047
$MHCII^+$ $CD163^+$ macrophages	% of $CD45^+$	5.13	**1.05E-03**						0.004	0.005			
	% of macrophages	8.59	**9.20E-06**			0.050	0.0013		0.002	<0.001		0.043	
$MHCII^+$ $CD163^-$ macrophages	% of $CD45^+$	16.90	**6.43E-10**		<0.001	<0.001	<0.001		<0.001	<0.001			
	% of macrophages	22.24	**3.89E-12**		<0.001	<0.001	<0.001		<0.001	<0.001	0.042	<0.001	
$MHCII^-$ $CD14^-$ macrophages	% of $CD45^+$	6.03	**2.90E-04**				0.0028		0.041	0.001			
	% of macrophages	5.25	**8.74E-04**				0.015		0.021	0.004			
$MHCII^-$ $CD14^+$ macrophages	% of $CD45^+$	8.76	**7.35E-06**			0.005	<0.001		0.045	<0.001		0.008	
	% of macrophages	8.95	**5.78E-06**			0.004	<0.001		0.012	<0.001		0.007	

Supplementay table 9: Statistical description of macrophage subsets by gene expression performed using v test after PCA analysis.

	MHCII$^-$ CD14$^+$		MHCII$^+$ CD163$^-$		MHCII$^+$ CD163$^+$	
	**v.test**	**p.value**	**v.test**	**p.value**	**v.test**	**p.value**
IL10			-3.636	0.0003		
CSF1	2.326	0.020			-2.509	0.012
VEGFA			-3.369	0.001	2.282	0.023
IL8			-3.572	0.0004		
IL1B			-3.435	0.001		
LYZ			-3.300	0.001		
ARG1			-3.330	0.001		
CCL2	2.214	0.027	-2.721	0.007		
MPEG1					3.062	0.002
S100A8			-3.045	0.002		
FCGR2B					2.675	0.007
IL4I1			2.727	0.006		
MMP14	1.991	0.047	-1.968	0.049		
CCL5			-2.773	0.006		
CD80			-2.669	0.008		
CD274			-2.227	0.026		
AIF1					1.970	0.049
MMP9					-1.991	0.046
IL6					-2.167	0.030
NOS2					-2.398	0.016
